# In Vivo Blood Kinetics and Transcript Integrity of Three mRNA–Lipid Nanoparticle Vaccines in Humans

**DOI:** 10.1021/acsnano.6c09226

**Published:** 2026-10-15

**Authors:** Stephen J. Kent, Shiyao Li, Thakshila H. Amarasena, Arnold Reynaldi, Michael G. Leeming, Jennifer A. Juno, Adam K. Wheatley, Georgia Deliyannis, Dale I. Godfrey, Terry Nolan, Colin W. Pouton, Miles P. Davenport, Yi Ju

**Affiliations:** † Department of Microbiology and Immunology, Peter Doherty Institute for Infection and Immunity, 2281 The University of Melbourne, Melbourne, Victoria 3000, Australia; ‡ Melbourne Sexual Health Centre and Department of Infectious Diseases, Alfred Hospital and Central Clinical School, Monash University, Melbourne, Victoria 3000, Australia; § Olivia Newton-John Cancer Research Institute, and School of Cancer Medicine, 607542 La Trobe University, Heidelberg, Victoria 3084, Australia; ∥ School of Science, RMIT University, Melbourne, Victoria 3000, Australia; ⊥ Infection Analytics Program, Kirby Institute for Infection and Immunity, 7800 University of New South Wales, Sydney, New South Wales 2052, Australia; # Melbourne Mass Spectrometry and Proteomics Facility, the Bio21 Molecular Science and Biotechnology Institute, The University of Melbourne, Parkville, Victoria 3010, Australia; ∇ Drug Delivery, Disposition and Dynamics, Monash Institute of Pharmaceutical Sciences, 208050 Monash University, Parkville, Victoria 3052, Australia; †† Department of Infectious Diseases, Peter Doherty Institute for Infection and Immunity, 2281 The University of Melbourne, Melbourne, Victoria 3000, Australia

**Keywords:** PEGylated lipid nanoparticle, mRNA degradation, SARS-CoV-2, vaccine pharmacokinetics, PEG immunogenicity, nanoparticle clearance and biodistribution, bio−nano interactions

## Abstract

mRNA–lipid nanoparticle (LNP) vaccines are detectable in human blood after vaccination, but platform-specific differences in systemic persistence and transcript integrity remain poorly defined. We analyzed serial blood samples from 73 participants receiving Moderna mRNA-1273 (three formulations), Pfizer/BioNTech BNT162b2, or an investigational receptor-binding domain (RBD) mRNA vaccine (three different doses). Using droplet digital polymerase chain reaction (ddPCR) assays, we quantified total and long-range linked (“intact”) vaccine mRNA, and we measured vaccine-specific ionizable lipids by liquid chromatography–mass spectrometry (LC–MS). Across platforms, mRNA decay was fastest for mRNA-1273, intermediate for BNT162b2, and slowest for the RBD vaccine, with ionizable lipid decay following the same rank order. Notably, intact spike mRNA declined 2-fold faster after mRNA-1273 than BNT162b2 vaccination. Kinetics modeling revealed platform-dependent coupling of mRNA and lipid kinetics: intact mRNA tracked closely with SM-102 for mRNA-1273, whereas ALC-0315 persisted longer than intact mRNA for BNT162b2. A ten-fragment linkage ddPCR panel spanning the spike transcript showed lower linkage toward 3′-proximal regions that mirrored the administered mRNA-1273 formulation. Together, these data provide a quantitative framework for benchmarking mRNA–LNP platform kinetics and transcript integrity in humans.

## Graphical abstract

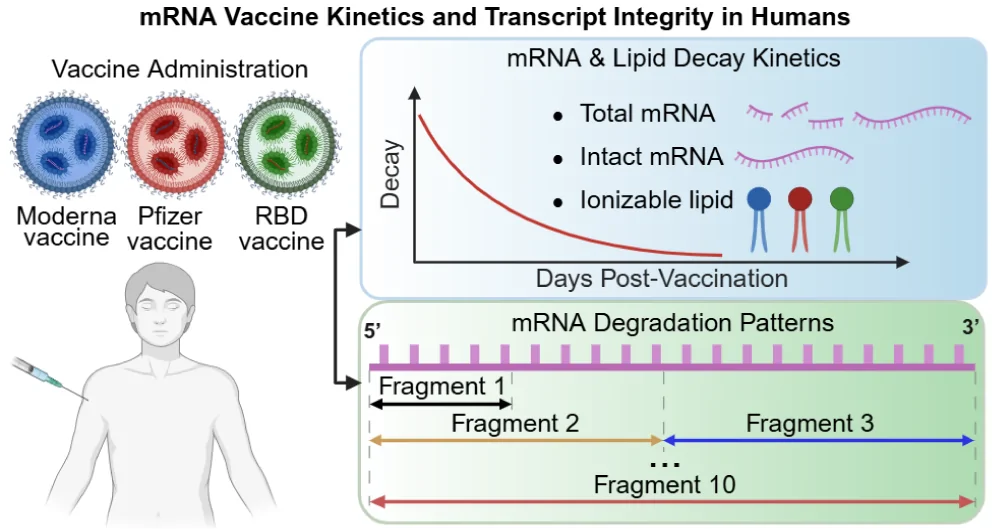

## Introduction

mRNA vaccines and therapeutics are transforming modern medicine, yet the *in vivo* distribution, persistence, and degradation of mRNA–lipid nanoparticle (LNP) formulations remain incompletely understood.^1^ The two most widely used SARS-CoV-2 mRNA vaccines are serially updated versions of the Moderna mRNA-1273 (Spikevax) and Pfizer/BioNTech BNT162b2 (Comirnaty) (hereafter referred to as “Moderna” and “Pfizer”, respectively). In parallel, multiple next-generation mRNA vaccines are in development and clinical testing, including candidates encoding shorter spike segments such as the receptor-binding domain (RBD).^2^ Notably, Moderna has generally shown modestly higher immunogenicity and effectiveness than Pfizer, but is administered at a higher dose (currently 50 μg vs 30 μg) and is associated with greater reactogenicity.^3,4^ The molecular and biophysical factors contributing to these platform-level differences remain poorly defined.

Defining how vaccine components behave in human blood provides a practical window into *in vivo* kinetics of mRNA–LNPs that would otherwise require sampling of human tissues. We and others have shown that both vaccine mRNA and vaccine-specific ionizable lipids can be quantified in serial blood samples for weeks following vaccination.^5–7^ These studies enabled detailed kinetic modeling and identified clinical and immunological correlates of circulating vaccine mRNA levels and decay.^7^ Although antipolyethylene glycol (PEG) antibodies were initially hypothesized to accelerate clearance, given that PEG-lipids are present in mRNA–LNPs, no significant association with faster decay was observed. Instead, higher peak concentrations of circulating vaccine mRNA and ionizable lipid correlated with stronger boosting of anti-PEG antibody levels.^7^ However, a head-to-head comparison of mRNA decay, lipid persistence, and transcript integrity across different mRNA–LNP vaccine platforms in humans has not been reported.

Improving mRNA stability and limiting degradation could, in principle, enhance vaccine performance by increasing the duration and magnitude of antigen expression. In small animal models, increasing mRNA stability or reducing degradation can increase antigen expression and improve vaccine responses.^8^ Beyond vaccines, systemically administered mRNA–LNP therapeutics have entered clinical trials, including candidates for *in vivo* gene editing and immunotherapy.^9–11^ Because intravenously administered mRNA–LNPs necessarily transit through and interact with blood before reaching target tissues, understanding the fate of LNP-formulated mRNA in human blood is increasingly important for improving the efficacy and safety of emerging mRNA–LNP therapeutics.

A key unresolved question is how LNP-formulated mRNA loses integrity *in vivo*. Naked mRNA is rapidly degraded by extracellular RNases,^12^ whereas LNP encapsulation provides substantial protection. Nonetheless, several aspects of transcript integrity in human blood remain unclear: (i) the extent to which integrity loss reflects fragmentation already present in the administered formulation versus postvaccination *in vivo* degradation; (ii) how rapidly long-range mRNA integrity is lost in blood over time; and (iii) whether specific transcript regions are disproportionately vulnerable to degradation.

Here, we analyzed serial blood samples from participants receiving licensed Moderna or Pfizer spike mRNA vaccines or an investigational SARS-CoV-2 RBD mRNA–LNP vaccine (MIPSCo-mRNA-RBD-1; NCT05272605; hereafter referred to as “mRNA–RBD”) evaluated in a phase I clinical trial^2^ (Figure 1A). We compared the blood kinetics of total mRNA, long-range linked mRNA (as a measure of transcript integrity), vaccine-specific ionizable lipid, and anti-PEG and anti-spike antibodies across platforms (Figure 1B). To map integrity loss along the transcript, we further developed a panel of linkage droplet digital polymerase chain reaction (ddPCR) assays spanning the spike coding region. Together, these data provide a comprehensive, platform-level view of mRNA and lipid persistence and transcript integrity in human blood, establishing a quantitative framework for benchmarking mRNA–LNP formulations *in vivo*.

**Figure 1:**
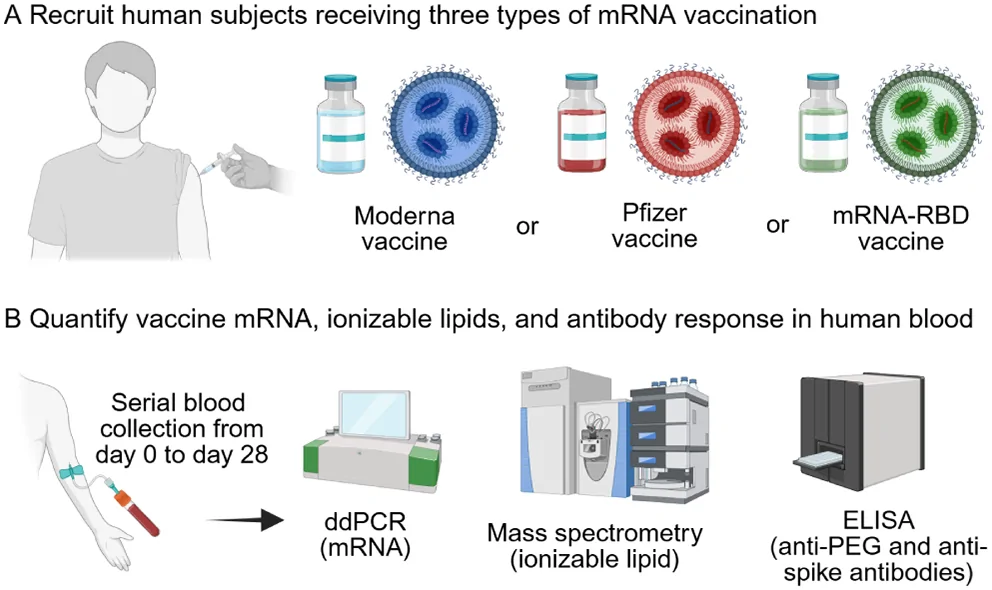
Schematic illustration of the workflow for comparative analysis of three mRNA vaccines in human blood. (A) Three cohorts of human participants were recruited who received either Moderna, Pfizer, or mRNA-RBD vaccination. (B) Serial blood samples were collected before and after vaccination and analyzed by ddPCR, mass spectrometry, and ELISA to quantify vaccine mRNA, ionizable lipid, and antibody response (anti-PEG and anti-spike), respectively. Created with BioRender.com.

## Results and Discussion

### SARS-CoV-2 mRNA–LNP Vaccine Clinical Studies

We analyzed longitudinal blood samples from 73 participants intramuscularly vaccinated with Moderna mRNA-1273 (*n* = 29; mRNA dose = 50 μg; type = bivalent ancestral + BA.1, bivalent ancestral + BA.5, or monovalent XBB.1.5), Pfizer/BioNTech BNT162b2 (*n* = 12; mRNA dose = 30 μg; type = ancestral), or an investigational mRNA–RBD vaccine (*n* = 32; mRNA dose = 10, 20, or 50 μg; type = B.1.351 RBD) (details of participants and vaccination are provided in Table S1). A total of 327 samples (median 3, range 2–15 samples per participant) were serially collected prevaccination (day 0) and from 4 h to day 28 postvaccination or later. Sampling schedules differed across studies: Moderna and Pfizer cohorts included multiple early time points, whereas the mRNA–RBD trial sampled primarily on days 0, 7, and 28. Plasma (with EDTA or heparin anticoagulation) and serum samples were aliquoted and stored at −80 °C.

### Vaccine mRNA Exhibits Platform-Specific Decay Kinetics

Using reverse transcription (RT)-ddPCR assays, vaccine mRNA was detectable in blood after vaccination and declined over time across all three platforms (Figure 2A–C, Figure S1A–C). All prevaccination samples were undetectable (0 copies μL^–1^) for vaccine mRNA. In the Moderna cohort, where early sampling was most frequent, total mRNA typically peaked at days 1–2 (median 1) postvaccination at a peak concentration of 0.001–0.529 ng mL^–1^ (median, 0.095) (Figure 2A; Figure S1A). At days 6–7 postvaccination, circulating mRNA concentrations were not significantly different between Moderna and Pfizer (Figure 2D), whereas mRNA–RBD levels were 35- and 109-fold lower than Moderna and Pfizer, respectively. Mixed-effects modeling of postpeak decay demonstrated platform-dependent decay rates (Figure 2E), with Moderna exhibiting the fastest mRNA decline (median 0.389 day^–1^), Pfizer an intermediate decline (0.250 day^–1^), and mRNA–RBD the slowest (0.123 day^–1^).

**Figure 2:**
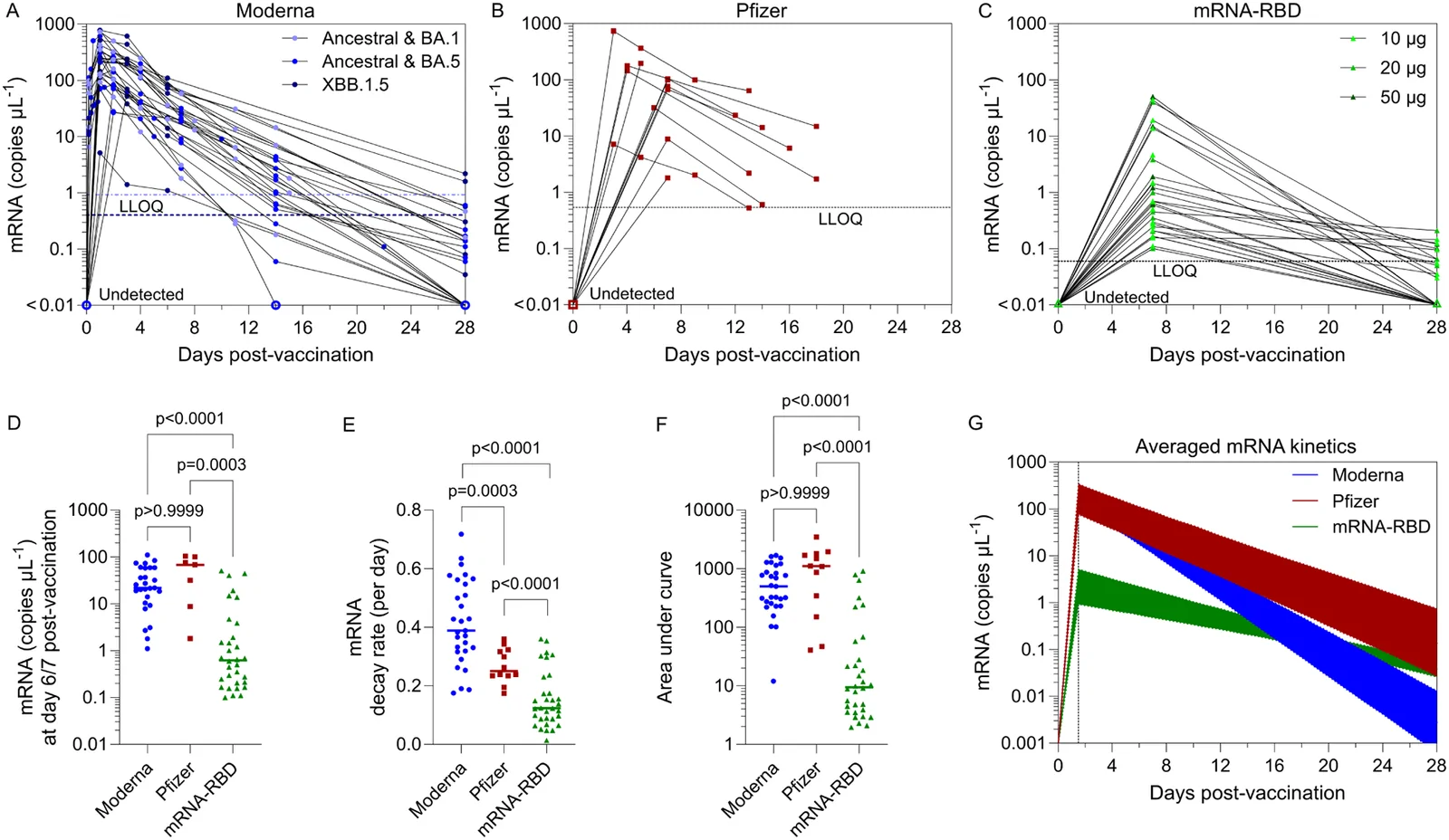
Comparison of *in vivo* vaccine mRNA kinetics in human blood following three types of SARS-CoV-2 mRNA vaccination (Moderna, Pfizer, or mRNA-RBD). (A–C) Longitudinal vaccine mRNA concentrations (copies μL^–1^) in human blood from seven cohorts receiving either (A) Moderna bivalent ancestral + BA.1, bivalent ancestral + BA.5, or monovalent XBB.1.5; (B) Pfizer; or (C) mRNA-RBD at 10, 20, or 50 μg doses. The lower limit of quantification (LLOQ; dashed line) was determined from linear standard curves (Figure S1D–G). In panel A, two LLOQs are shown: 0.4 copies μL^–1^ for Moderna XBB.1.5 (dark blue dashed line) and 0.93 copies μL^–1^ for Moderna bivalent vaccines (light blue dashed line). Undetected samples (0 copies μL^–1^) were plotted with open symbols. (D–G) Comparison of (D) mRNA concentration at days 6–7 postvaccination, (E) postpeak mRNA decay rates, (F) postpeak area under the curve (AUC) of mRNA kinetics in blood, and (G) averaged mRNA kinetics across donors among the three vaccine types. In (D–F), each dot represents one participant, and the horizontal line indicates the median. In (G), averaged mRNA kinetics are shown as mean predictions from the best-fit linear model, with shaded regions indicating the 95% confidence interval bounds. Statistical analysis was performed using the nonparametric Kruskal–Wallis test with Dunn’s multiple comparisons in (D, F) and the likelihood ratio test in (E).

To enable cross-platform comparison of circulating mRNA exposure despite differences in sampling density, we assumed that circulating vaccine mRNA peaked at day 1.5 and subsequently followed a log-linear decay. This assumption was supported by the densely sampled Moderna cohort, in which total mRNA generally peaked at days 1–2 (mean of day 1.5). Because the Pfizer and mRNA–RBD cohorts were not sampled near the assumed day 1.5 peak, their peak concentrations were not directly observed and were instead back-extrapolated from later measurements using the fitted decay trajectories. Under this model, we calculated the postpeak area under the curve (AUC) from the fitted decay trajectories. We found that postpeak mRNA exposure was not significantly different between Moderna and Pfizer (median AUC 498.4 vs 1104.7), whereas mRNA–RBD exposure was markedly lower (median AUC 9.4), corresponding to ∼53-fold and ∼117-fold lower exposure than Moderna and Pfizer, respectively (Figure 2F,G). Within the Moderna cohort, postpeak AUC did not differ significantly among the BA.1- and BA.5-bivalent and XBB.1.5 formulations (Figure S2A). Similarly, mRNA–RBD AUCs were not significantly different across the 10, 20, and 50 μg dose groups, with substantial interindividual variability (Figure S2D).

### Ionizable Lipid Exposure and Clearance Differ across Vaccines

We next quantified each platform’s vaccine-specific ionizable lipid by targeted liquid chromatography–mass spectrometry (LC–MS) (SM-102 for Moderna, ALC-0315 for Pfizer, and DLin-MC3-DMA for mRNA–RBD; LNP formulations in Table 1). All three ionizable lipids were negative (below the lower limit of quantification, LLOQ) in prevaccination samples but became readily detectable at early postvaccination time points and declined thereafter (Figure 3A–C, Figure S3A–C). Analytical sensitivity and platform specificity were supported by lipid standard curves (Figure S3D–G), representative chromatograms (Figure S3H–K), and cross-platform negative controls (Figure S4).

**Table 1:**
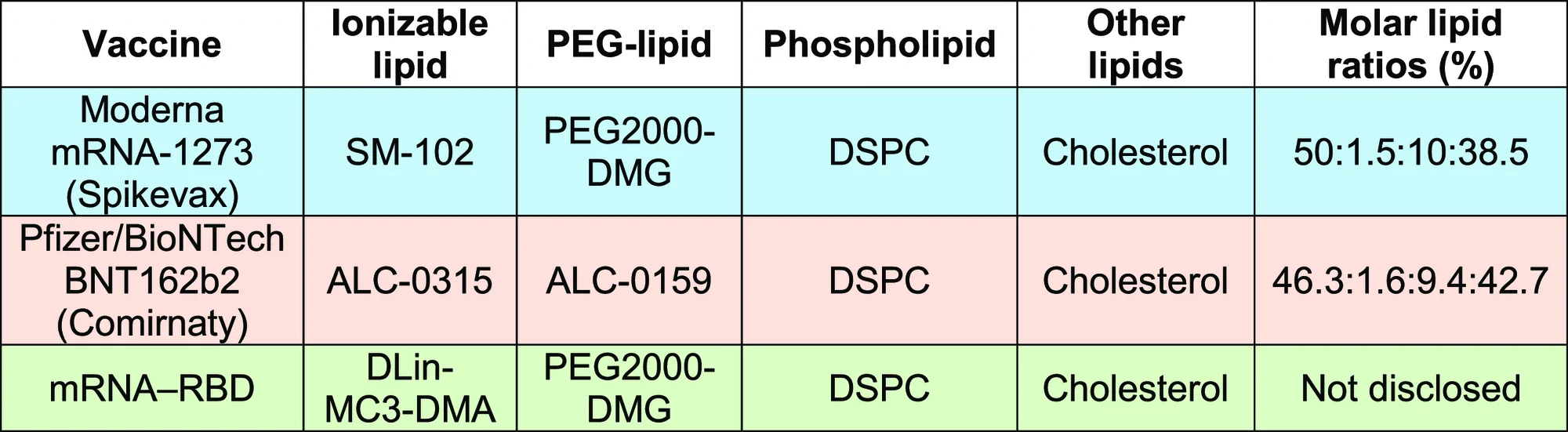
Four Lipid Components in the LNP Formulation of Moderna mRNA-1273 (Spikevax), Pfizer/BioNTech BNT162b2 (Comirnaty), and the mRNA–RBD SARS-CoV-2 mRNA Vaccines^a^

a Molar lipid ratios (%) are presented in the order of ionizable lipid: PEG-lipid: phospholipid: cholesterol.

**Figure 3:**
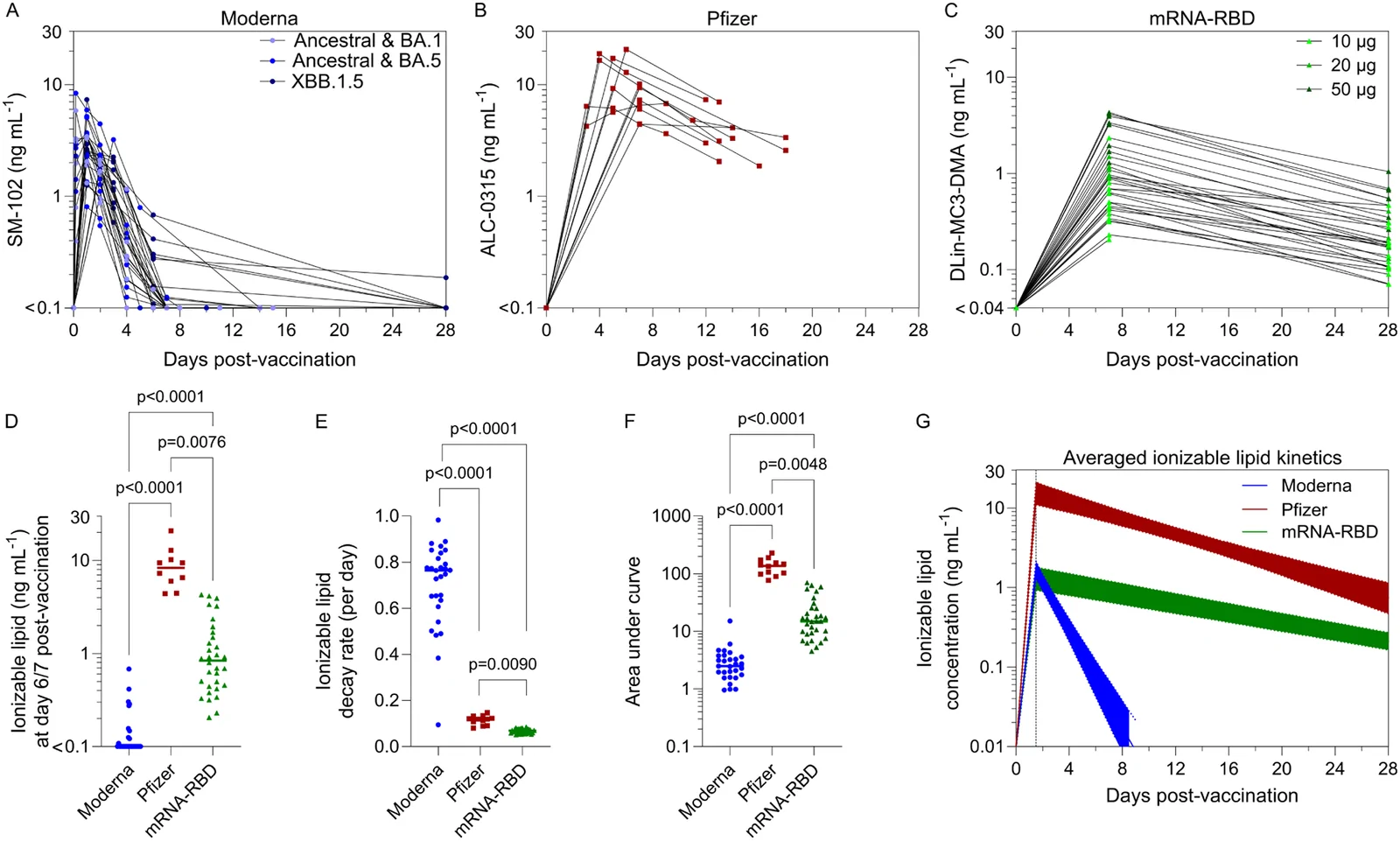
Comparison of *in vivo* kinetics of ionizable lipids (SM-102, ALC-0315, DLin-MC3-DMA) in human blood following Moderna, Pfizer, or mRNA-RBD vaccination, respectively. (A–C) Longitudinal ionizable lipid concentrations (ng mL^–1^) in human blood from seven cohorts who received either (A) Moderna bivalent ancestral + BA.1, bivalent ancestral + BA.5, or monovalent XBB.1.5 (formulated with SM-102); (B) Pfizer (formulated with ALC-0315); or (C) mRNA-RBD (formulated with DLin-MC3-DMA) vaccination at 10, 20, or 50 μg doses. (D–G) Comparison of (D) ionizable lipid concentration at days 6–7 postvaccination, (E) postpeak ionizable lipid decay rates, (F) postpeak AUC of ionizable lipid kinetics in blood, and (G) averaged ionizable lipid kinetics across donors among the three vaccine types. In (D–F), each dot represents one participant, and the horizontal line indicates the median. In (G), averaged ionizable lipid kinetics are shown as mean predictions from the best-fit linear model, with shaded regions indicating the 95% confidence interval bounds. Statistical analysis was performed using the nonparametric Kruskal–Wallis test with Dunn’s multiple comparisons in (D, F) and the likelihood ratio test in (E).

In the Moderna cohort, SM-102 peaked at 4 h to day 2 postvaccination at a peak concentration of 0.81–8.40 (median 2.63) ng mL^–1^ (Figure 3A). In the Pfizer cohort, the earliest postvaccination time points were days 3–5, with ALC-0315 concentrations peaking at 4.24 to 18.89 (median 12.90) ng mL^–1^ (Figure 3B). At days 6–7 postvaccination, circulating ALC-0315 concentrations (median 8.33 ng mL^–1^) were highest in the Pfizer cohort, followed by DLin-MC3-DMA (median 0.84 ng mL^–1^) in the mRNA–RBD cohort, whereas SM-102 in the Moderna cohort was often below the LLOQ (0.1 ng mL^–1^) (Figure 3D).

Consistent with the rank order observed for vaccine mRNA decay, lipid decay was fastest for Moderna (median 0.765 day^–1^), intermediate for Pfizer (median 0.120 day^–1^), and slowest for mRNA–RBD (median 0.069 day^–1^) (Figure 3E). To compare overall circulating exposure across platforms, ALC-0315 lipid exposure was greatest in the Pfizer cohort (median AUC 136.5), corresponding to a 54.6- and 9.2-fold higher exposure than SM-102 in the Moderna cohort (median AUC 2.5) and DLin-MC3-DMA in the mRNA–RBD cohort (median AUC 14.9), respectively (Figure 3F,G). Within the Moderna cohort, SM-102 exposure was slightly higher after the XBB.1.5 booster (median AUC 4.0) than after the bivalent boosters (median AUC 1.9 for ancestral + BA.1 and 2.1 for ancestral + BA.5) (Figure S2B). Within the mRNA–RBD trial, DLin-MC3-DMA exposure increased with dose, with median AUCs of 9.1, 15.8, and 51.8 for 10, 20, and 50 μg, respectively (Figure S2E).

### Integrity of Circulating Vaccine mRNA Decreases Faster after Moderna Than Pfizer Vaccination

The total vaccine mRNA measurements above rely on short amplicons (113 bp for Moderna and Pfizer; 134 bp for mRNA–RBD; Tables S2, S3) and therefore cannot distinguish long transcripts from shorter degraded fragments. To quantify long-range transcript integrity, we applied a linkage RT-ddPCR assay in which two widely separated targets on the same spike transcript are detected within individual droplets. An excess of double-positive droplets above that expected from random co-occupancy was used to infer that the two target regions of the mRNA molecule remain physically linked across the intervening region. For simplicity, we refer to these molecules as “intact mRNA”; throughout this manuscript, “intact” means intact across the specific length of sequence assayed and does not indicate that the entire transcript is full length. The linkage assays spanned 1599 bp for Moderna and 1404 bp for Pfizer (Tables S4, S5), corresponding to ∼42% and ∼37%, respectively, of the 3819-bp spike coding sequence. Because circulating mRNA–RBD vaccine mRNA levels were low at the earliest available time point (day 7), intact mRNA could not be reliably quantified for the mRNA–RBD cohort.

Across early postvaccination samples, the fraction of intact spike mRNA was broadly similar between Moderna and Pfizer, generally ∼10–25% of total vaccine mRNA detected (Figure 4A,B). In the Moderna cohort, the intact fraction was highest at 4 h to day 1 postvaccination (7.4–19.7%, median 13.7%) and then declined over time (Figure 4A). In the Pfizer cohort, sampling began at days 3–4, when the intact fraction was 12.3–26.8% (median 24.2%), and it subsequently decreased (Figure 4B). Combining total vaccine mRNA concentrations with intact fractions, we calculated intact mRNA concentrations, which showed log–linear decay kinetics (Figure 4C,D). By days 6–7 postvaccination (among participants with a sample available at day 6 or 7 ), intact mRNA remained above the LLOQ in 20 of 27 Moderna participants (74%) and 6 of 7 Pfizer participants (86%), with median intact mRNA concentrations of 2.1 and 8.3 copies μL^–1^, respectively. In the Pfizer cohort, two additional participants without a day 6–7 sample had intact mRNA above the LLOQ at day 9 or day 18, bringing the overall proportion with detectable intact mRNA to 89% (8 of 9 participants).

**Figure 4:**
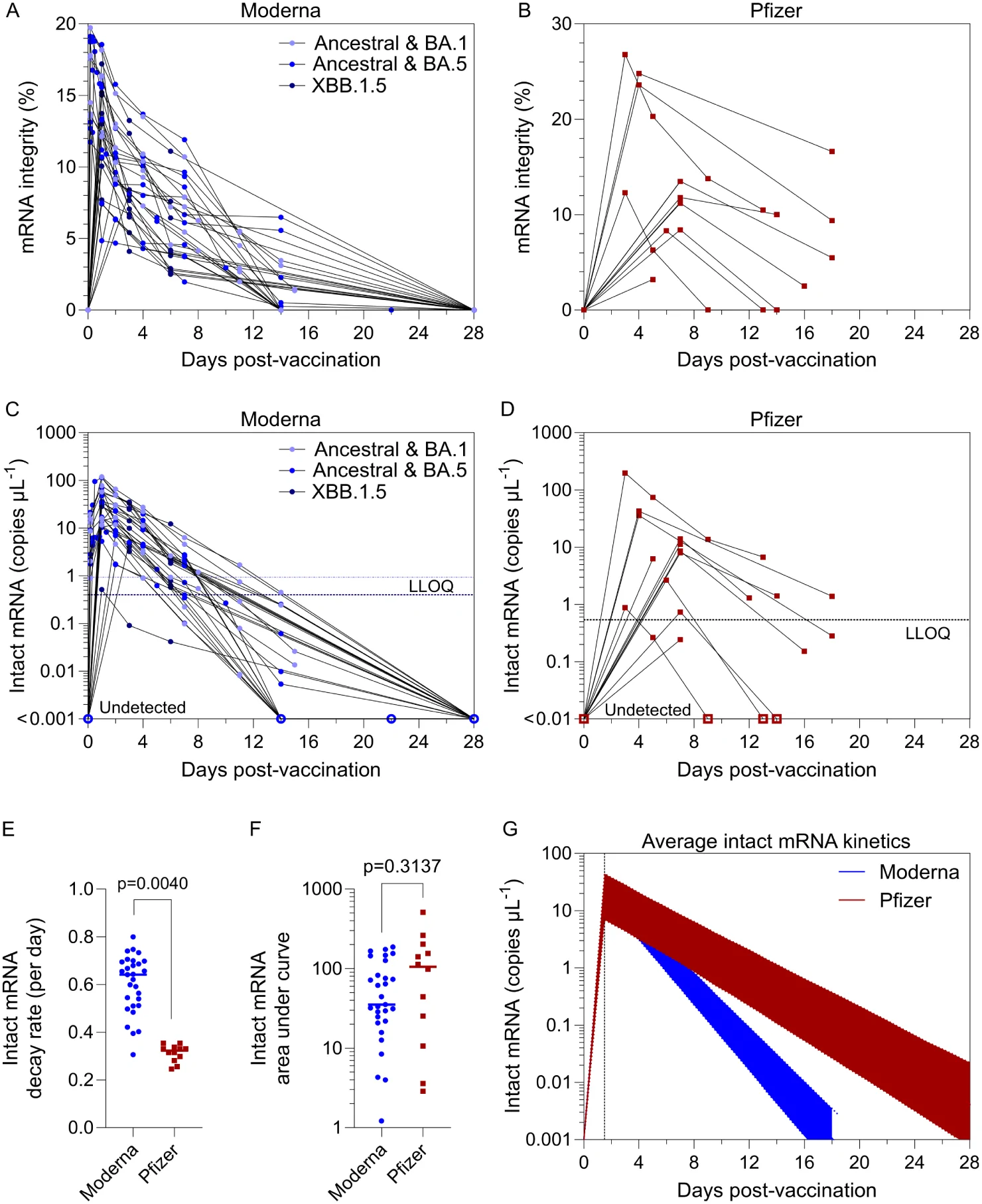
Comparison of *in vivo* vaccine mRNA integrity in human blood between Moderna and Pfizer vaccines. (A, B) Longitudinal vaccine mRNA integrity in human blood from four cohorts who received either (A) Moderna bivalent ancestral + BA.1, bivalent ancestral + BA.5, or monovalent XBB.1.5; or (B) Pfizer vaccination. (C, D) Longitudinal intact vaccine mRNA concentration (copies μL^–1^) in human blood from the four cohorts. The LLOQ (shown as a dashed line) was determined based on the linear standard curves of vaccine mRNA (Figure S1D–F). In panel C, two LLOQs are shown: 0.4 copies μL^–1^ for Moderna XBB.1.5 (dark blue dashed line) and 0.93 copies μL^–1^ for Moderna bivalent vaccines (light blue dashed line). Undetected samples (0 copies μL^–1^) were plotted with open symbols. (E–G) Comparison of (E) postpeak intact mRNA decay rates, (F) postpeak AUC of intact mRNA kinetics in blood, and (G) averaged intact mRNA kinetics across donors between the two vaccine types. In (E, F), each dot represents one participant, and the horizontal line indicates the median. In (G), averaged intact mRNA kinetics are shown as mean predictions from the best-fit linear model, with shaded regions indicating the 95% confidence interval bounds. Statistical analysis was performed using the nonparametric Mann–Whitney *U* test in (F) and the likelihood ratio test in (E).

Mixed-effects modeling of postpeak decay showed that intact mRNA declined ∼2-fold faster after Moderna than Pfizer vaccination (median 0.64 vs 0.32 day^–1^; Figure 4E). Consistent with these kinetics, modeled postpeak intact mRNA exposure (AUC) showed a higher median in the Pfizer cohort than in the Moderna cohort (median 105.4 vs 35.4), although this difference was not statistically significant due to substantial interindividual variability (Figure 4F,G). Intact mRNA exposure was similar across the three Moderna booster formulations (Figure S2C).

### Platform-Specific mRNA-Ionizable Lipid Kinetic Coupling

To enable direct comparison across analytes and vaccine platforms, we normalized each readout to its postvaccination peak (set to 100%) and plotted best-fit decay trajectories for total mRNA, intact mRNA, and ionizable lipid (Figure 5A), together with the corresponding half-life estimates (Figure 5B). In the Moderna cohort, intact mRNA and SM-102 declined with closely aligned trajectories (blue dashed lines in Figure 5A) and similar half-lives (Figure 5B), consistent with our prior observations,^7^ suggesting that circulating intact mRNA largely remains associated with ionizable lipid in blood. In contrast, in the Pfizer cohort, ALC-0315 concentrations declined more slowly than intact mRNA (red dashed lines in Figure 5A) and exhibited a longer half-life (Figure 5B), indicating a longer persistence of ionizable lipid in circulation than intact mRNA. In the mRNA–RBD cohort, intact mRNA could not be quantified reliably, but DLin-MC3-DMA and total mRNA showed the slowest decay and longest half-lives among the three platforms (Figure 5A,B).

**Figure 5:**
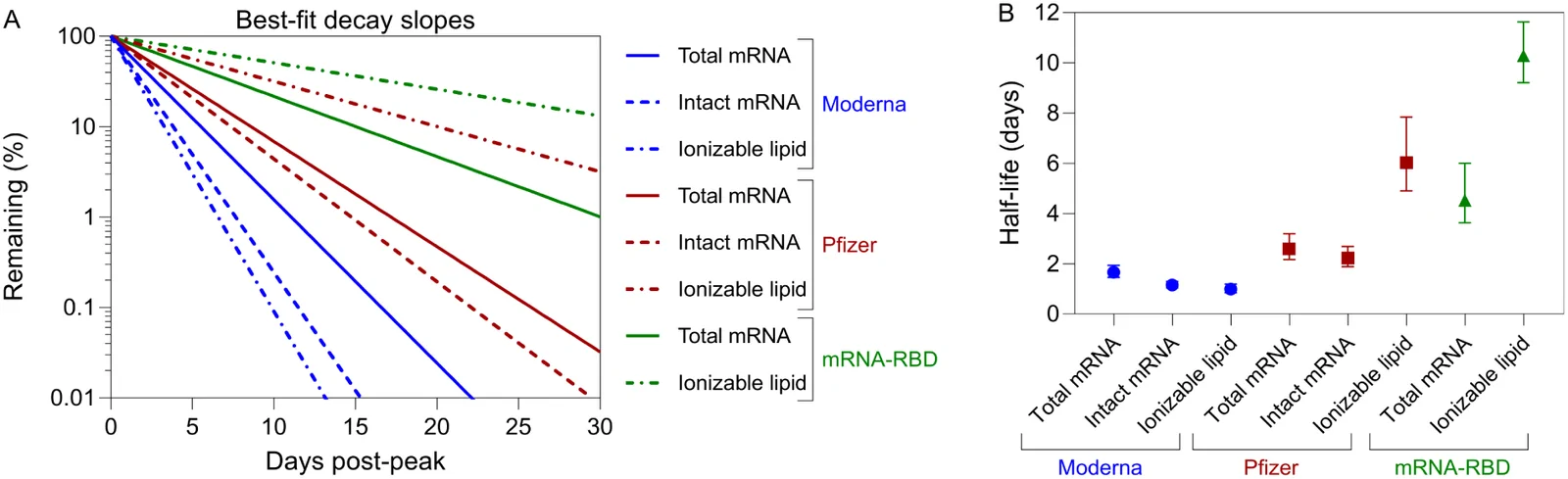
Comparison of *in vivo* decay kinetics of vaccine mRNA and ionizable lipids in human blood following Moderna, Pfizer, or mRNA-RBD vaccination. (A) Best-fit decay slopes of total mRNA, intact mRNA, and ionizable lipids across the three vaccines. The response at the peak time point for each parameter was normalized to 100%, and the percentage change over time illustrates the decline estimated from the best-fit linear model. (B) Half-life of total mRNA, intact mRNA, and ionizable lipids from the three vaccines, shown as the mean with upper and lower bounds of 95% confidence intervals calculated across multiple donors in each cohort.

The biological processes underlying the vaccine mRNA decay and ionizable lipid decay are not equivalent. Loss of intact mRNA may reflect molecular degradation as well as clearance, whereas the decline in ionizable lipid concentration in plasma likely reflects predominantly clearance, although hydrolysis and other metabolic transformations may also contribute. The divergence between intact mRNA and ALC-0315 in the Pfizer cohort may have several possible explanations, including the presence of empty or partially degraded LNPs from original vaccine products, nuclease-mediated degradation of mRNA following its exposure from destabilized LNPs postvaccination, and dissociation of ALC-0315 followed by association with plasma components. Since our assays do not determine the particle-level colocalization of the detected mRNA and lipid, these interpretations remain speculative but generate testable hypotheses for future studies.

### Anti-PEG and Anti-Spike Antibody Responses across Vaccine Platforms

The PEG-lipid component of mRNA–LNP vaccines can induce anti-PEG antibodies. We and others have previously reported higher anti-PEG IgG and IgM responses following Moderna than Pfizer vaccination.^13–15^ However, anti-PEG responses induced by experimental mRNA–LNP vaccines used in clinical trials, such as the mRNA–RBD vaccine studied here, remain unclear. We quantified plasma anti-PEG IgG and IgM titers by ELISA using our established method.^14^ Cohort immunization context differed: the Pfizer cohort received a prime (first-ever) SARS-CoV-2 mRNA vaccination, whereas the Moderna and mRNA–RBD cohorts received booster vaccinations in participants with prior COVID-19 vaccination (Table S1). Postvaccination samples were collected at day 28 for the Moderna and mRNA–RBD cohorts, and between days 12–70 for the Pfizer cohort.

Prior to vaccination, anti-PEG IgG (end-point titer >1:10) was detectable in 24/29 (83%), 10/12 (83%), and 19/26 (73%) participants in the Moderna, Pfizer, and mRNA-RBD cohorts, respectively (Figure 6A). Pre-existing anti-PEG IgM was detectable in 28/29 (97%), 7/12 (58%), and 11/26 (42%) participants in the Moderna, Pfizer, and mRNA–RBD cohorts, respectively (Figure 6B). Following vaccination, anti-PEG antibody titers increased in a platform- and isotype-dependent manner. In the Moderna cohort, both anti-PEG IgG and IgM were boosted at day 28, with mean fold changes of 2.0 (range 1–6.7) and 5.8 (range 1–33.6), respectively (Figure 6C,D). In contrast, after Pfizer vaccination, anti-PEG IgG showed no significant increase (mean fold change 1.1, range 0.8–1.4), whereas anti-PEG IgM increased significantly (mean fold change 1.6, range 1–5.1). In the mRNA–RBD cohort, more modest increases were observed, with mean fold changes of 1.2 (range 0.9–2.1) for anti-PEG IgG and 1.8 (range 0.8–11.9) for anti-PEG IgM. Overall, anti-PEG IgM showed a greater boost than anti-PEG IgG across the three vaccine platforms, and Moderna induced larger increases in both anti-PEG IgG and IgM than Pfizer or mRNA–RBD. Within the Moderna cohort, anti-PEG responses were similar across the three Moderna booster formulations (Figure S5A,B), and no clear dose-dependent trend was observed across the mRNA–RBD dose groups (Figure S5C,D).

**Figure 6:**
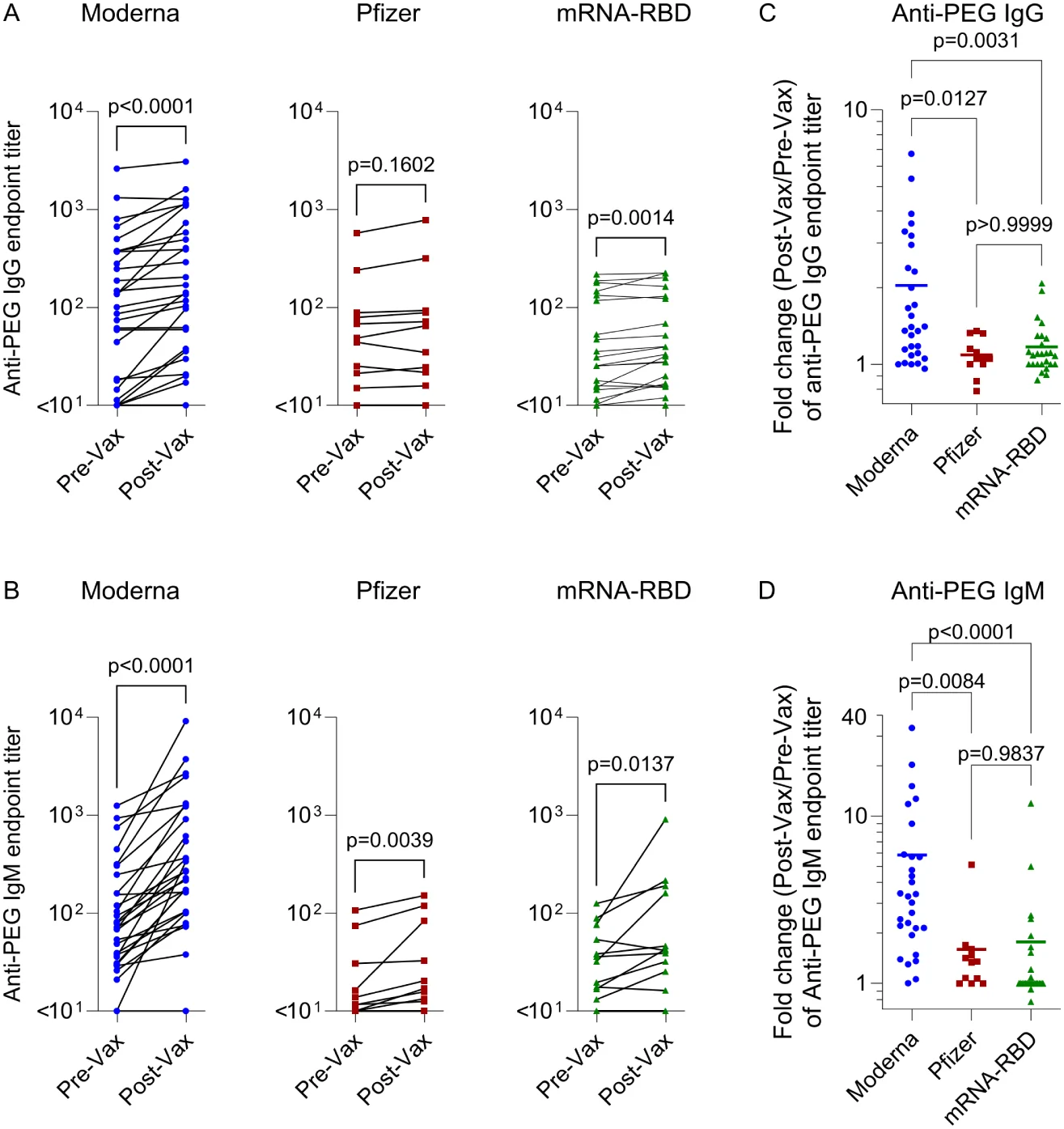
Comparison of anti-PEG antibody levels in human blood before and after Moderna, Pfizer, or mRNA-RBD vaccination. (A, B) Comparison of plasma anti-PEG IgG and IgM end point titers before vaccination (Pre-Vax) and after vaccination (Post-Vax) for the three vaccine types. (C, D) Cross-comparison of fold changes (Post-Vax/Pre-Vax) in anti-PEG IgG and IgM end point titers among the three vaccine types. In (C, D), each dot represents one participant, and the horizontal line indicates the mean. Statistical analysis was performed using the nonparametric Wilcoxon matched-pairs signed-rank test in (A, B) and the nonparametric Kruskal–Wallis test with Dunn’s multiple comparisons in (C, D).

The observation that Moderna induced greater anti-PEG responses than Pfizer raises the question of whether this is driven by dose (50 μg vs 30 μg) or LNP composition.^13^ Interestingly, we observed no dose-dependent increase in PEG immunogenicity for the mRNA–RBD vaccine across the 10, 20, and 50 μg dose groups, suggesting that dose alone may not fully explain the observed differences. Although the mRNA–RBD and Moderna vaccines utilize the same PEG-lipid (PEG2000-DMG), they elicited different anti-PEG responses and contain different ionizable lipids (DLin-MC3-DMA and SM-102, respectively). Since ionizable lipids possess adjuvant properties that can promote antigen immunogenicity,^16^ these observations raise the hypothesis that ionizable lipid chemistry may contribute to anti-PEG antibody induction. Larger clinical cohorts and animal studies directly comparing matched formulations will be required to test this hypothesis.

Vaccine immunogenicity was confirmed by significant increases in plasma anti-spike IgG titers after vaccination in all three cohorts (Figure S6A–C). The magnitude of anti-spike IgG boosting did not differ across the three Moderna formulations (Figure S6D), and anti-spike IgG fold changes were similar across the 10, 20, and 50 μg mRNA–RBD dose groups (Figure S6E), indicating no significant formulation- or dose-dependent effects within the tested ranges. Notably, anti-spike antibodies may be less informative for the mRNA-RBD vaccine than for the Moderna and Pfizer vaccines, as the former encodes only the RBD, which is less than 20% of spike protein and presents a far more limited range of target antigens.

### Degradation Patterns of Moderna Vaccine mRNA

The prototype linkage RT-ddPCR assay above measures integrity across a single long span (37–42%) of spike mRNA, but it cannot determine whether integrity loss is biased toward particular regions of the transcript. To map degradation patterns along the spike transcript, we developed a ten-fragment linkage RT-ddPCR panel, in which each fragment is defined by two widely separated targets that are codetected within the same droplet (Figure 7A). The panel includes fragments shorter and longer than the original linkage assay (corresponding to fragment 6 in Figure 7A) and covers both the 5′-proximal and 3′-proximal regions of the transcript. We applied this panel to serial plasma samples from six Moderna recipients (three BA.1 bivalent and three BA.5 bivalent), selected based on longitudinal sample availability.

**Figure 7:**
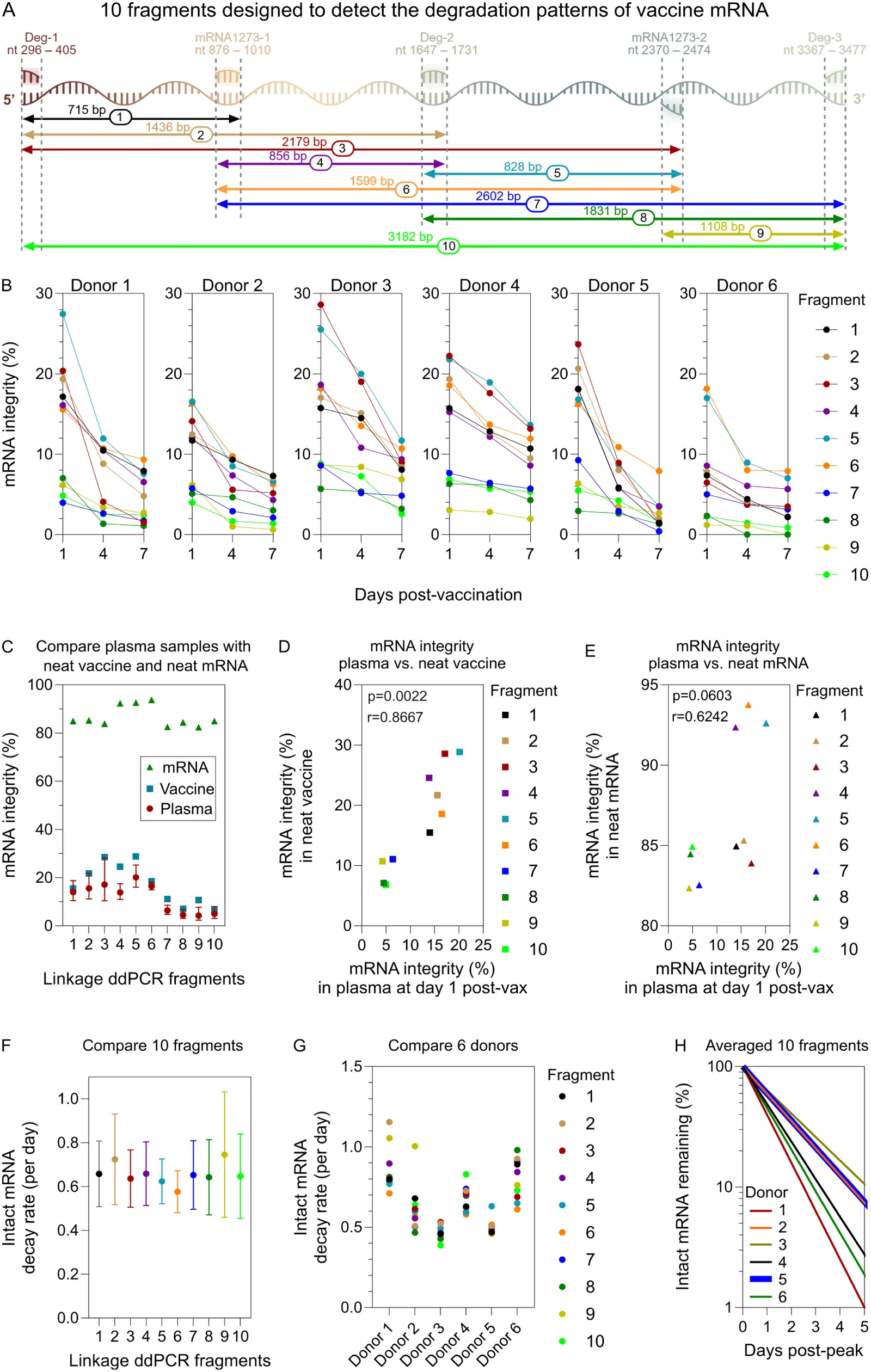
*In vivo* degradation patterns of Moderna vaccine mRNA in human blood evaluated using ten two-primer fragments in a duplex PCR assay. (A) Schematic illustration of the ten two-primer fragments, each targeting two regions of the Moderna vaccine mRNA sequence in the duplex ddPCR assay to assess degradation patterns of vaccine mRNA. Created with BioRender.com. (B) Vaccine mRNA integrity in plasma from six subjects at days 1, 4, and 7 post-Moderna vaccination (three subjects received the bivalent ancestral + BA.1 vaccine and three received the bivalent ancestral + BA.5 vaccine) assessed using the ten fragments. (C) Comparison of mRNA integrity across the 10 fragments in plasma samples (day 1 postvaccination), neat Moderna vaccine, and synthetic Moderna vaccine mRNA. (D, E) Spearman correlation analysis between mRNA integrity in plasma at day 1 postvaccination and mRNA integrity in (D) neat Moderna vaccine or (E) synthetic Moderna vaccine mRNA. (F, G) Comparison of intact mRNA decay rates across (F) the ten fragments and (G) the six subjects. (H) Best-fit decay slopes of intact mRNA across six donors, with each data point representing the average decay rate calculated from ten individual fragments. For each donor, decay rates were estimated separately for each fragment, and the mean of these ten fragment-specific rates was used to represent the donor-level decay slope. As the decay slopes of donors 2 and 5 overlap, the curve of donor 5 was plotted with higher thickness than that of donor 2 to improve readability. In (C, F), mRNA integrity (%) and decay rates in plasma samples are shown as the mean with upper and lower bounds of 95% confidence intervals calculated across six donors.

Intact (linked across the assayed span) spike mRNA was detectable for all ten fragments in all six subjects over time (Figure 7B). At day 1 postvaccination, fragments nearest the 3′ end of the transcript (fragments 7–10) showed lower linkage (mean integrity 5.6%, range 1.2–9.3%) than midtranscript fragments (fragments 4–6, mean integrity 17.6%, range 8.6–27.5%) or 5′-proximal fragments (fragments 1–3, mean integrity 16.6%, range 6.5–28.6%). Importantly, the day-1 integrity profile in plasma closely mirrored that measured in the administered vaccine product: the relative abundance of linked mRNA across fragments at day 1 (red circles in Figure 7C) significantly correlated with fragment linkage in the original Moderna formulation (blue squares in Figure 7C,D). As a reference, synthetic spike mRNA, synthesized based on the putative Moderna vaccine mRNA sequence, was largely intact across all ten fragments (green triangles in Figure 7C, mean integrity 86.7%, range 82.4–93.7%) and did not show a significant correlation with the fragment-by-fragment profile observed in blood (Figure 7E). Together, these data suggest that (i) the pattern of fragment integrity detected in blood at day 1 largely reflects the formulation’s starting integrity profile; (ii) there was minimal further degradation between vaccination and day 1 postvaccination, when mRNA–LNPs travel *in vivo* into the blood; (iii) the integrity loss of vaccine product is biased toward the 3′-proximal regions of the transcript. Interestingly, the longest (near full-length) fragment (fragment 10) displayed comparatively low linkage at day 1 (mean integrity 5.4%, range 2.3–8.8%), a notable observation given that full-length transcripts are expected to contribute most directly to spike expression. Vaccine immunogenicity was nonetheless confirmed by significant boosting of anti-spike IgG after Moderna vaccination (Figure S6A,D).

To assess whether mRNA degrades in a region-specific manner while circulating in the human blood *in vivo*, we tracked each fragment over the first week postvaccination (Figure 7B). All ten linked fragments declined over time, but the decay rates were similar across fragments (mean 0.65, range 0.58–0.72 day^–1^; Figure 7F), suggesting no positional “hotspot” of transcript loss (or protection) *in vivo*. In contrast, decay rates clustered by individual (Figure 7G): fragment slopes were more similar within a subject than between subjects. Across six subjects and ten fragments, ∼92% of the variance in decay rates was attributable to subject-to-subject differences, whereas ∼8% was attributable to fragment identity (based on mixed-effects modeling). We then plotted the mean decay rate across the 10 fragments for each individual against time (days postpeak), revealing substantial interpersonal differences (Figure 7H).

## Conclusions

The rational improvement of mRNA vaccines and therapeutics requires a precise understanding of how mRNA–LNP components behave *in vivo* in humans, particularly regarding their systemic distribution, persistence, and transcript integrity over time. In this study, we performed a head-to-head comparison of three mRNA–LNP vaccine platforms: Moderna, Pfizer, and mRNA–RBD vaccine, across 73 participants and 327 serial blood samples. By quantifying total vaccine mRNA, long-range linked (“intact”) mRNA, platform-specific ionizable lipids, and antibody responses, we identified distinct platform-level differences. While peak circulating mRNA concentrations were broadly comparable between Moderna and Pfizer, their decay kinetics diverged significantly: Moderna displayed the fastest clearance, Pfizer an intermediate rate, and mRNA–RBD the slowest. Notably, while ionizable lipid kinetics followed this same rank order, the total lipid exposure (AUC) was highest for Pfizer. Furthermore, our linkage RT-ddPCR analysis revealed that intact mRNA declined more rapidly following Moderna vaccination than Pfizer vaccination, highlighting platform-specific differences in the stability of long-range transcript integrity in circulation. Finally, while all platforms robustly boosted anti-spike IgG, Moderna induced significantly larger boosts in anti-PEG IgG and IgM than the Pfizer or mRNA–RBD vaccines.

These findings have important implications for the clinical application and evaluation of mRNA platforms. Conceptually, the detection of vaccine-derived mRNA and lipid in the blood likely represents a “spillover” event from the injection site or draining lymph nodes. Assuming an average adult blood volume of 5 L, we estimate that a mean of 1.4% (range 0.01–5.3%) of the administered Moderna mRNA dose was present in systemic circulation at day 1 postvaccination. By days 6–7, only a minute fraction (mean 0.15% of the administered mRNA dose) remains detectable. In contrast, higher proportions of the administered mRNA doses from Pfizer and mRNA–RBD vaccines remained detectable in blood at later time points (mean 1.9% and 0.5% at days 6–7, respectively), likely driven by their slower decay rates relative to Moderna. While the clinical impact of systemic vaccine distribution remains to be fully defined, circulating components may distribute to secondary lymphoid tissues, such as the spleen, potentially influencing immune responses or reactogenicity at distant sites. Although our study was not powered to robustly link circulating vaccine levels with side effects, these pharmacokinetic measurements provide a framework for future studies investigating whether systemic vaccine exposure is associated with safety and reactogenicity outcomes.

The divergence in “intact” mRNA stability, specifically the 2-fold longer half-life of intact Pfizer mRNA compared to Moderna, is of particular interest. While our measurements are restricted to blood, it is plausible that similar stability profiles exist within lymph nodes. Theoretically, prolonged antigen availability could amplify humoral immunity by synchronizing antigen delivery with the developing germinal center response, a mechanism shown to enhance antibody titers in animal models.^17^

The platform-specific differences in mRNA stability and ionizable lipid kinetics may be influenced by the biomolecular coronas acquired by LNPs *in vivo*. Following intramuscular administration, LNPs encounter proteins and lipids first in interstitial and lymphatic fluids and subsequently in plasma upon entering the circulation. Adsorption of these biomolecules generates a biomolecular corona whose composition is shaped by formulation properties and biofluid composition.^18^ These coronas may affect LNP stability, alter the exposure of the mRNA to extracellular nucleases, and promote the exchange of lipid components with plasma lipoproteins.^19^ Pre-existing anti-PEG antibodies may further modify the corona by binding to surface-exposed PEG and recruiting complement proteins, potentially modulating immune recognition and clearance.^20^ Future studies characterizing biomolecular corona formation on mRNA–LNP vaccines could help determine whether and how these coronas influence mRNA–LNP stability and clearance *in vivo*.

At the transcript level, our comprehensive suite of linkage RT-ddPCR assays further advances the characterization of mRNA degradation by mapping integrity across the entire spike transcript. We identified a distinct positional bias in degradation: the 3′ end of the spike mRNA was most prone to loss, both in the vaccine formulation and in early postvaccination blood samples. Remarkably, as little as 2.3% of the spike mRNA appeared intact across our largest fragment (spanning 3182 bp, or ∼83% of the coding region) in early blood samples. This indicates that the vast majority of circulating mRNA exists as fragments. Future studies are required to determine whether loss of 3′-region integrity causes reduced vaccine immunogenicity. If this hypothesis is confirmed, strategies to stabilize the 3′ region and increase the proportion of full-length transcripts could represent a tangible approach to enhancing the potency and efficiency of future mRNA vaccines.

Broadly, typical vaccine evaluations prioritize immune end points and reactogenicity, often overlooking the physical pharmacokinetics of the vaccine components themselves. We advocate for a more balanced evaluation framework that includes the systemic distribution, persistence, and mRNA integrity of the vaccine. Our approach, initially driven by PCR-based mRNA detection, now expanded to LC–MS-based lipid detection and linkage ddPCR-enabled mRNA integrity mapping, offers a granular view of vaccine behavior *in vivo*. Recent work has detected PEG in human plasma samples,^21^ suggesting it should be possible to measure the PEG-lipids of mRNA–LNP vaccines in plasma. Similar analyses could be attempted with other vaccine modalities.

We acknowledge several limitations in our study that should be explored in future studies. The mRNA–RBD studies were limited since the cohort was sampled only at days 0, 7, and 28, and our modeling may not therefore capture accurate postvaccination peak and clearance rates. It is clear now from our sampling of Moderna and Pfizer cohorts that sampling within the first week postvaccination is most valuable, and future human mRNA-LNP vaccine studies can now be designed to yield accurate peak and clearance rates. Additionally, the heterogeneity in participants’ prior infection and vaccination history may introduce variability, though this reflects the real-world landscape of current vaccine administration. While this is the largest study of its kind, larger cohorts would allow for more robust correlation analyses between blood vaccine kinetics, immunogenicity, and reactogenicity. Technically, while our linkage RT-ddPCR provides the most comprehensive map of transcript integrity to date, it does not offer single-nucleotide resolution; deep sequencing remains challenging due to the abundance of host RNA in blood. Furthermore, the ten-fragment linkage RT-ddPCR analysis was restricted to plasma samples from six Moderna recipients selected based on longitudinal sample availability. This small sample size could limit the generalizability of the observed positional integrity patterns and subject-to-subject variability. Studies in larger independent cohorts will be required to validate these findings. Finally, our analysis was restricted to blood; future studies examining biodistribution in draining lymph nodes, spleen, and mucosal sites would provide a more complete anatomical picture.

Overall, we document and compare the *in vivo* kinetics of SARS-CoV-2 mRNA–LNP vaccine components from three vaccine platforms in humans. By establishing a quantitative framework to benchmark systemic persistence and transcript integrity, this work provides critical insights to guide the rational design of next-generation mRNA vaccines with improved stability, immunogenicity, and safety.

## Methods

### Ethics Statement

The study protocols were approved by the University of Melbourne Human Research and Ethics Committee (approval no. 2056689), and all associated procedures were carried out in accordance with the approved guidelines. The investigational RBD mRNA–LNP vaccine clinical study was approved by the Royal Melbourne Hospital Human Research Ethics Committee and was conducted under the Clinical Trial Notification (CTN) Scheme (CTN ID: 04968-1) administered by the Australian Therapeutic Goods Administration and registered at www.clinicaltrials.gov (NCT05272605). All participants provided written informed consent in accordance with the Declaration of Helsinki.

### Participant Recruitment and Sample Collection

We recruited 29 participants who received a Moderna mRNA-1273 Spikevax booster vaccination and 12 participants who received a Pfizer/BioNTech BNT162b2 vaccination. In addition, we analyzed plasma samples from 32 participants enrolled in a previously reported investigational RBD mRNA–LNP vaccine clinical trial.^2^ Demographic characteristics and SARS-CoV-2 vaccination history for all 73 participants are summarized in Table S1. Whole blood was collected into tubes containing sodium heparin or ethylenediaminetetraacetic acid (EDTA) anticoagulant, or into serum-separating tubes. Plasma and serum were aliquoted and stored at −80 °C.

### Quantification of COVID-19 Vaccine mRNA

COVID-19 vaccine mRNA in plasma was quantified by reverse transcription droplet digital polymerase chain reaction (RT-ddPCR) as previously reported.^7^ Total RNA was isolated from 140 μL of EDTA-anticoagulated plasma or serum using the QIAmp RNA Extraction Kit (Qiagen). RNA (10 μL) was reverse transcribed using Superscript III Reverse Transcriptase according to the manufacturers’ instructions (Invitrogen). Primers and probes were designed based on the putative vaccine mRNA sequences (Tables S2 and S3) and synthesized by Integrated DNA Technologies (USA). These primer/probe sets were specific for the codon-modified vaccine mRNA sequences and did not amplify wild-type SARS-CoV-2 spike (S)-gene.

ddPCR was performed on a QX200 system (Bio-Rad). Each 24-μL reaction contained 12 μL of 2× ddPCR Supermix for Probes (no dUTP, Bio-Rad), 5 μL of cDNA, 3 μL of primer/probe mix, and 4 μL of nuclease-free water. Droplets were generated using DG8 droplet generator cartridges (Bio-Rad) and a QX200 droplet generator (Bio-Rad). PCR was performed on a C1000 Touch thermal cycler (Bio-Rad) with the following program: 95 °C for 10 min; 40 cycles of 94 °C for 30 s and 60 °C for 60 s; and 98 °C for 10 min, with a ramp rate of 2 °C/s. Droplets were read on a QX200 droplet reader (Bio-Rad), and data were analyzed using QuantaSoft software v1.7.4.

Vaccine mRNA from the original vaccine product was used as a positive control and to set the positive droplet threshold and to generate linear standard curves. Vaccine was spiked into negative human plasma (collected prevaccination), followed by RNA extraction and reverse transcription as described above. cDNA was serially diluted 10-fold in nuclease-free water and analyzed by ddPCR. Copy numbers of the vaccine mRNA in the ddPCR reaction were used to derive the copy number per μL of plasma. The linear PCR standard curve (Figure S1D–G) was derived in GraphPad Prism 10 with relative weighting (weighting by 1/*y* ^2^) and used to convert mRNA concentration from copies μL^–1^ to ng mL^–1^.

### Quantification of Ionizable Lipids

Ionizable lipids (SM-102, ALC-0315, and DLin-MC3-DMA) were quantified by targeted liquid chromatography–mass spectrometry (LC–MS) as previously reported.^7^ For sample preparation, 50 μL of diluted plasma (90% plasma, prepared by mixing 45 μL of neat plasma with 5 μL of phosphate-buffered saline (PBS)) was extracted with 500 μL of 1-butanol/methanol (1:1, v/v). Then, samples were vortexed for 10 s and sonicated for 60 min in a water bath at 20 °C. To generate linear standard curves, the original vaccine product (with known ionizable lipid concentration) was serially diluted in PBS, and 5 μL of diluted vaccine was spiked into 45 μL of negative human plasma (collected prevaccination), followed by the same extraction procedure as described above. Samples were subsequently centrifuged at 16,000*g* for 20 min, and 100 μL of the resulting supernatant was transferred to glass LC autosampler vials (Phenomenex, USA) for analysis.

Samples were analyzed using a Shimadzu 8050 triple quadrupole mass spectrometer coupled to a Shimadzu Nexera X2 liquid chromatography system. Sample components were separated on an Agilent RRHD Eclipse Plus C18 column (2.1 × 100 mm, 1.8 μm; Agilent Technologies, USA) over a 15 min gradient using mobile phase A (6:4 water/acetonitrile containing 10 mM ammonium acetate and 5 μM medronic acid) and mobile phase B (9:1 isopropanol/acetonitrile containing 10 mM ammonium acetate). The solvent gradient was programmed as follows [time (min), *B* (%)]: [0, 5], [2, 5], [10, 98], [12, 98], [12.5, 5], [15, 5]. The solvent flow rate was maintained at 0.3 mL min^–1^, and the eluent was diverted to waste for the first 5 min. The autosampler chamber and column oven were maintained at 10 and 40 °C, respectively.

Analytes were ionized by electrospray ionization in positive ion mode and detected using a multiple reaction monitoring (MRM) strategy (Table S7). The nebulizing gas flow rate was 2 L min^–1^, and heating and drying gas flow rates were 10 L min^–1^. The interface temperature and voltage were maintained at 375 °C and 4 kV, respectively. Collision energies and MRM parameters were optimized using the in-built Shimadzu LabSolutions MRM optimization tool.

Raw data were processed using Skyline (v23.1). Analytes were quantified by integrating peak areas from the summed MRM transitions. Standard curves (Figure S3D–G) were fitted using weighted linear regression (1/*x* ^2^) and used to calculate the ionizable lipid concentrations.

### Linkage ddPCR to Measure Relative Levels of Intact mRNA

We performed linkage RT-ddPCR as originally developed by Hanna et al. to quantify vaccine mRNA integrity in human breast milk^22^ and as subsequently adapted by us to quantify vaccine mRNA integrity in human plasma.^7^ Linkage RT-ddPCR uses a duplex ddPCR design in which two widely separated regions of the same spike mRNA molecule are detected simultaneously using probes labeled with different fluorophores (FAM and HEX). Droplets that are double-positive (FAM+HEX) indicate that both target regions were present within the same partition and therefore remain physically linked on the same RNA molecule across the assayed span (i.e., “intact” across that region). In contrast, droplets that are single-positive (FAM-only or HEX-only) are consistent with fragmented mRNA species that contain only one of the two target regions.

Linkage duplex ddPCR reactions were prepared and run using the same droplet generation, thermal cycling, droplet reading, and analysis workflow described above for total vaccine mRNA quantification, except that the linkage primer/probe sets (Tables S4, S5) were combined in a single duplex reaction (FAM and HEX). Data were analyzed in QuantaSoft v1.7.4. QuantaSoft was used to compute the linkage number (linked copies), defined as the excess number of double-positive droplets above that expected from random co-occupancy of unlinked targets (i.e., correcting for chance colocalization).

Percent linkage (the “intact” fraction across the assayed span) was calculated as:

$$\%linkage=\frac{linkage\;number}{\left(\frac{copies_{FAM}+copies_{HEX}}{2}\right)}\times 100$$

where copies_FAM_ and copies_HEX_ are the single-target concentrations reported for each channel in QuantaSoft.

To map integrity patterns along the spike transcript, we expanded the original linkage assay into a ten-fragment linkage RT-ddPCR panel, using distinct duplex primer/probe pairs to quantify linkage across ten different transcript spans (Figure 7A; Table S6). Each fragment was analyzed by using the same duplex linkage workflow described above. As a reference control, synthetic spike mRNA was produced by Messenger Bio (Australia) based on the putative Moderna vaccine mRNA sequence and assayed alongside vaccine and plasma samples.

### Quantification of Anti-PEG Antibody

Anti-PEG IgG and IgM were quantified by enzyme-linked immunosorbent assay (ELISA) as previously described.^14^ Briefly, eight-arm PEG-NH_2_ (40 kDa; 200 μg/mL; JenKem Technology, USA) in PBS was coated onto MaxiSorp 96-well plates (Nunc, Denmark) for 18 h at 4 °C. Plates were washed four times with PBS and blocked with 5% (w/v) skim milk in PBS for 22 h. Serially diluted human plasma samples (prepared in 5% skim milk in PBS) were added in duplicate and incubated for 1 h at 22 °C. Plates were washed twice with 0.1% 3-[(3-cholamidopropyl)-dimethylammonio]-1-propanesulfonate (CHAPS; Sigma-Aldrich, USA) in PBS and then four times with PBS before adding horseradish peroxidase (HRP)-conjugated antihuman IgG (Dako Agilent, USA; 1:20,000 dilution) or HRP-conjugated antihuman IgM (Jackson ImmunoResearch Laboratories, USA; 1:10,000 dilution) for 1 h at 22 °C. Plates were washed as above and developed with 3,3′,5,5′-tetramethylbenzidine (TMB) liquid substrate (Sigma-Aldrich, USA). Reactions were stopped with 0.16 M H_2_SO_4_, and absorbance was read at 450 nm. End point titers were defined as the reciprocal plasma dilution yielding a signal 2-fold above background, calculated in GraphPad Prism 10 using a fitted curve (4-parameter logistic regression) and reported as the mean of duplicates. Background signals were determined by applying plasma (1:10 dilution in 5% skim milk) to non-PEG-coated wells and processing in parallel.

### Quantification of Anti-Spike IgG

Plasma IgG binding to the ancestral SARS-CoV-2 spike protein was measured by ELISA following an established protocol.^7^ Briefly, 96-well MaxiSorp plates were coated overnight at 4 °C with recombinant HexaPro^23^ spike protein (2 μg mL^–1^). Plates were washed twice with PBS containing 0.05% Tween-20 and once with PBS, then blocked with PBS containing 1% fetal calf serum (FCS) for 1 h at 22 °C. Plasma samples were serially diluted 4-fold (1:100 to 1:1,638,400), added in duplicate, and incubated for 2 h at 22 °C. Following incubation, plates were washed four times with PBS containing 0.05% Tween-20 and then twice with PBS. Bound IgG was detected using HRP-conjugated antihuman IgG antibody (Agilent, USA; 1:20,000 dilution). Plates were developed with TMB substrate, the reaction was stopped with 0.16 M H_2_SO_4_, and absorbance was read at 450 nm. End point titers were calculated using GraphPad Prism 10 as the reciprocal plasma dilution yielding a signal 2-fold above background using a fitted curve (4 parameter logistic regression) and reported as the mean of duplicates. Background signals were determined by averaging the signals from all plasma samples at a dilution of 1:1,638,400.

### Estimating Decay Rates and Postpeak Area Under the Curve (AUC)

Concentrations of vaccine mRNA and ionizable lipid in plasma were assumed to reach a single postvaccination peak followed by log–linear decay. For cross-platform modeling, day 1.5 postvaccination was used as the common reference peak time, based on the day 1–2 peaks observed in the densely sampled Moderna cohort. Days postpeak were therefore calculated relative to this reference time. For the Pfizer and mRNA–RBD cohorts, in which samples were not available during this early period, concentrations at the assumed peak were back-extrapolated from the available postpeak measurements under the same kinetic assumption.

Decay rates of mRNA and lipid species in plasma were estimated using a linear mixed-effects model with *days postpeak* and *vaccine type* (Moderna, Pfizer, mRNA–RBD) as fixed effects. Subject-specific random intercepts and slopes were included to account for between-individual variability in peak concentrations and decay rates. To represent exponential decay, response variables were log-transformed prior to analysis. Observations below the lower limit of quantitation (LLOQ) were treated as left censored. Random effects were assumed to follow a bivariate normal distribution, and residual errors were modeled as independent and normally distributed. Model fitting was performed using maximum likelihood estimation implemented in the *lmec* package in *R* (version 4.4.0). Differences in decay rates among vaccine types were assessed using likelihood ratio tests.

For each group, the fitted postpeak concentration was described using the estimated intercept and slope from the log–linear decay model. The fitted concentration at the assumed peak was obtained by back-extrapolating this fitted curve to day 1.5. The postpeak AUC was calculated by integrating the fitted decay curve from the assumed peak time (day 1.5) to day 28. No 95% confidence interval bound was calculated for the interval from day 0 to day 1.5; the 95% confidence interval bounds for day 1.5 to day 28 were derived from the estimated decay curve with back extrapolation. Accordingly, the reported AUCs are model-derived estimates rather than noncompartmental AUCs calculated directly from observed concentrations.

### Statistical Analysis

Decay rates of total mRNA (Figure 2E), ionizable lipid (Figure 3E), and intact mRNA (Figure 4E) were compared using likelihood ratio tests in R (v4.4.0). Comparisons across the three vaccine platforms for mRNA concentration at days 6–7 (Figure 2D), postpeak mRNA AUC (Figure 2F), ionizable lipid concentration at days 6–7 (Figure 3D), ionizable lipid AUC (Figure 3F), and fold changes in anti-PEG IgG and IgM titers (Figure 6C,D) were performed using the nonparametric Kruskal–Wallis test with Dunn’s multiple comparisons in GraphPad Prism 10. Intact mRNA AUC was compared between Moderna and Pfizer (Figure 4F) using the nonparametric Mann–Whitney *U* test in GraphPad Prism 10. Paired comparison of anti-PEG IgG and IgM titers pre- versus postvaccination (Figure 6A,B) and anti-spike IgG titers pre- versus postvaccination (Figure S6A–C) was performed using the nonparametric Wilcoxon matched-pairs signed-rank test in GraphPad Prism 10. Associations between mRNA integrity in plasma at day 1 postvaccination and mRNA integrity in neat Moderna vaccine or synthetic Moderna vaccine mRNA (Figure 7D,E) were assessed using the nonparametric Spearman correlation in GraphPad Prism 10. Comparisons of AUCs for total mRNA, ionizable lipid, intact mRNA across Moderna formulations and across mRNA-RBD dose groups (Figure S2A–E), as well as fold changes in anti-PEG and anti-spike antibody titers across Moderna formulations or mRNA–RBD dose groups (Figure S5A–D and Figure S6D,E) were performed using the Kruskal–Wallis test with Dunn’s multiple comparisons in GraphPad Prism 10.

## Supplementary Material



## Notes

Demographic information and SARS-CoV-2 immunization history of participants receiving Moderna mRNA-1273, Pfizer/BioNTech BNT162b2, or mRNA–RBD vaccination; primer and probe sequences for total mRNA quantification, linkage RT-ddPCR analysis of mRNA integrity, and ten-fragment transcript degradation mapping; optimized multiple reaction monitoring parameters for SM-102, ALC-0315, and DLin-MC3-DMA detection; standard curves and longitudinal measurements of vaccine mRNA and ionizable lipid concentrations in plasma; comparisons of total mRNA, ionizable lipid, and intact mRNA exposure across Moderna formulations and mRNA–RBD dose groups; raw mass spectrometry data, representative chromatograms, and lipidomics specificity controls; comparisons of anti-PEG antibody boosting across vaccine formulations and dose groups; and anti-spike IgG responses before and after vaccination (PDF)
