# Supplementary material for: In Vivo Blood Kinetics and Transcript Integrity of Three mRNA–Lipid Nanoparticle Vaccines in Humans
Source: ACS Nano. 2026 Oct 15;20(42):28329–42. doi: 10.1021/acsnano.6c09226 (PMC13718610; doi:10.1021/acsnano.6c09226)
Supplement: Supplementary file 1 [file nn6c09226_si_001.pdf]

SUPPORTING INFORMATION

In Vivo Blood Kinetics and Transcript Integrity of Three mRNA–Lipid Nanoparticle Vaccines in Humans

Stephen J. Kent[1,2,], Shiyao Li[3,4], Thakshila H. Amarasena[1], Arnold Reynaldi[5], Michael G. Leeming[6], Jennifer A. Juno[1], Adam K. Wheatley[1], Georgia Deliyannis[1], Dale I. Godfrey[1], Terry Nolan[7], Colin W. Pouton[8], Miles P. Davenport[5], Yi Ju[3,4,*]*

[1]Department of Microbiology and Immunology, Peter Doherty Institute for Infection and Immunity, The University of Melbourne, Melbourne, Victoria 3000, Australia

[2]Melbourne Sexual Health Centre and Department of Infectious Diseases, Alfred Hospital and Central Clinical School, Monash University, Melbourne, Victoria 3000, Australia

[3]Olivia Newton-John Cancer Research Institute, and School of Cancer Medicine, La Trobe University, Heidelberg, Victoria 3084, Australia

[4]School of Science, RMIT University, Melbourne, Victoria 3000, Australia

[5]Infection Analytics Program, Kirby Institute for Infection and Immunity, University of New South Wales, Sydney, New South Wales 2052, Australia

[6]Melbourne Mass Spectrometry and Proteomics Facility, The Bio21 Molecular Science and Biotechnology Institute, The University of Melbourne, Parkville, Victoria 3010, Australia

[7]Department of Infectious Diseases, Peter Doherty Institute for Infection and Immunity, The University of Melbourne, Melbourne, Victoria 3000, Australia

[8]Drug Delivery, Disposition and Dynamics, Monash Institute of Pharmaceutical Sciences, Monash University, Parkville 3052, Victoria, Australia

Table S1. Demographic information and SARS-CoV-2 immunization history of 73 participants who received either Moderna mRNA-1273 (Spikevax), Pfizer/BioNTech BNT162b2 (Comirnaty), or mRNA–RBD SARS-CoV-2 mRNA vaccination.

Participant ID	Age	Sex	SARS-CoV-2 Vaccine history	Moderna Spikevax vaccination		
				Type	mRNA dose (μg)	Days since last COVID-19 vaccination
1	36	M	Pfizer (×3)	Ancestral & BA.1	50	401
2	28	F	Pfizer (×3)	Ancestral & BA.1	50	343
3	31	F	Pfizer (×3)	Ancestral & BA.1	50	424
4	39	M	AstraZeneca (×2) + Moderna (×1) + Pfizer (×1)	Ancestral & BA.1	50	191
5	61	M	AstraZeneca (×2) + Moderna (×1) + Novavax (×1)	Ancestral & BA.1	50	192
6	26	F	Pfizer (×3)	Ancestral & BA.1	50	346
7	59	F	AstraZeneca (×2) + Moderna (×1)	Ancestral & BA.5	50	412
8	65	F	AstraZeneca (×2) + Pfizer (×2)	Ancestral & BA.5	50	256
9	59	F	AstraZeneca (×2) + Moderna (×1)	Ancestral & BA.5	50	486
10	24	F	AstraZeneca (×2) + Moderna (×2)	Ancestral & BA.5	50	139
11	61	M	Novavax (×1) + Pfizer (×3)	Ancestral & BA.5	50	270
12	31	F	Pfizer (×2) + Moderna (×1)	Ancestral & BA.5	50	432
13	31	M	Pfizer (×3)	Ancestral & BA.5	50	433
14	30	F	Pfizer (×3)	Ancestral & BA.5	50	433
15	24	F	AstraZeneca (×3)	Ancestral & BA.5	50	419
16	28	M	Pfizer (×3)	Ancestral & BA.5	50	457

17	34	F	Pfizer (×1) + Moderna (×2)	Ancestral & BA.5	50	224
18	61	F	AstraZeneca (×2) + Moderna (×1)	Ancestral & BA.5	50	496
19	70	M	AstraZeneca (×2) + Moderna (×1)	Ancestral & BA.5	50	380
20	62	M	Pfizer (×3) + Moderna (×1)	XBB.1.5	50	355
21	33	F	Moderna (×3) + Pfizer (×1)	XBB.1.5	50	431
22	29	F	Pfizer (×3) + Moderna (×1)	XBB.1.5	50	508
23	29	F	Pfizer (×3) + Moderna (×1)	XBB.1.5	50	460
24	37	M	Pfizer (×3) + Moderna (×1)	XBB.1.5	50	460
25	33	F	Moderna (×2) + Pfizer (×1)	XBB.1.5	50	832
26	57	F	Pfizer (×3)	XBB.1.5	50	872
27	25	F	Covishield (×3) + Moderna (×1)	XBB.1.5	50	362
28	26	F	Sinopharm (×3)	XBB.1.5	50	740
29	30	F	AstraZeneca (×2) + Moderna (×2)	XBB.1.5	50	487
Summary	Median 33 (range 24−70)	F 69%	Median 3 vaccines (range 3−4)	BA.1 (n=6); BA.5 (n=13); XBB.1.5 (n=10)	50 µg (n=29)	Median 424 (range 139−872)

**Participant ID**	**Age**	**Sex**	**SARS-CoV-2 Vaccine history**	**Pfizer/BioNTech Comirnaty vaccination**		
				**Type**	**mRNA dose (µg)**	**Days since last COVID-19 vaccination**
30	55	F	None	Ancestral	30	N.A.
31	64	M	None	Ancestral	30	N.A.
32	23	F	None	Ancestral	30	N.A.
33	55	M	None	Ancestral	30	N.A.
34	67	F	None	Ancestral	30	N.A.
35	59	F	None	Ancestral	30	N.A.
36	60	M	None	Ancestral	30	N.A.
37	58	F	None	Ancestral	30	N.A.
38	23	F	None	Ancestral	30	N.A.
39	53	M	None	Ancestral	30	N.A.
40	35	F	None	Ancestral	30	N.A.
41	50	F	None	Ancestral	30	N.A.

Summary	Median 55 (range 23−67)	F 67%	None	Ancestral (n=12)	30 µg (n=12)	N.A.
**Participant ID**	**Age**	**Sex**	**SARS-CoV-2 Vaccine history**	**mRNA–RBD vaccination**		
				**Type**	**mRNA dose (µg)**	**Days since last COVID-19 vaccination**
42	61	M	AstraZeneca (×2) + Pfizer (×1)	B.1.351	10	111
43	48	F	Pfizer (×3)	B.1.351	10	99
44	48	F	Pfizer (×3)	B.1.351	10	165
45	34	F	AstraZeneca (×2) + Pfizer (×1)	B.1.351	10	148
46	61	F	AstraZeneca (×2) + Pfizer (×1)	B.1.351	10	169
47	59	M	AstraZeneca (×2) + Pfizer (×1)	B.1.351	10	151
48	53	F	AstraZeneca (×2) + Moderna (×1)	B.1.351	10	161
49	58	M	AstraZeneca (×2) + Pfizer (×1)	B.1.351	10	180
50	49	M	AstraZeneca (×2) + Moderna (×1)	B.1.351	10	194
51	50	F	Pfizer (×3)	B.1.351	10	192
52	35	M	Pfizer (×3)	B.1.351	10	196
53	63	F	AstraZeneca (×2) + Pfizer (×1)	B.1.351	10	188
54	31	M	Pfizer (×3)	B.1.351	10	156
55	37	F	Pfizer (×3)	B.1.351	10	187
56	35	F	Pfizer (×3)	B.1.351	10	248
57	48	M	Pfizer (×3)	B.1.351	10	213
58	57	M	AstraZeneca (×2) + Moderna (×1)	B.1.351	20	216
59	43	F	Pfizer (×3)	B.1.351	20	277
60	20	F	Pfizer (×3)	B.1.351	20	192
61	28	F	Pfizer (×3)	B.1.351	20	209
62	26	F	Pfizer (×3)	B.1.351	20	232
63	27	F	Pfizer (×3)	B.1.351	20	224
64	46	F	Pfizer (×3)	B.1.351	20	301

65	39	M	AstraZeneca (×2) + Pfizer (×1)	B.1.351	20	222
66	29	F	AstraZeneca (×2) + Moderna (×1)	B.1.351	50	241
67	40	M	Pfizer (×2) + Moderna (×1)	B.1.351	50	243
68	44	M	Pfizer (×2) + Moderna (×1)	B.1.351	50	278
69	21	M	AstraZeneca (×2) + Pfizer (×1)	B.1.351	50	270
70	45	M	Pfizer (×3)	B.1.351	50	322
71	31	F	Pfizer (×2) + Moderna (×1)	B.1.351	50	267
72	61	F	AstraZeneca (×2) + Moderna (×1)	B.1.351	50	302
73	55	F	AstraZeneca (×2) + Pfizer (×1)	B.1.351	50	288
Summary	Median 45 (range 20−63)	F 59%	3 vaccines (n=32)	B.1.351 (n=32)	10 μg (n=16); 20 μg (n=8); 50 μg (n=8)	Median 211 (range 99−322)

Table S2. Sequences of primer and probe designed specifically for the codon-modified vaccine mRNA sequences used in ddPCR assays to quantify Moderna mRNA-1273 (Spikevax) and Pfizer/BioNTech BNT162b2 (Comirnaty) vaccine mRNA levels in plasma (data shown in Figure 2A,B).

Primer or probe	Sequence	Target region
AMmRNA-F	5'-GAGCCTGCTGATCGTGAATAA-3'	nt 402 – 514 (113 bp)
AMmRNA-P	5'-/56-FAM/TCAAGGTGT/ZEN/GCGAGTTCCAGTTCT/3IABkFQ/-3'	
AMmRNA-R	5'-TCCAGCTCTTGTTGTTCTTGT-3'	

Table S3. Sequences of primer and probe designed specifically for the codon-modified vaccine mRNA sequences used in ddPCR assays to quantify mRNA–RBD vaccine mRNA levels in plasma (data shown in Figure 2C).

Primer or probe	Sequence	Target region
RBD-F	5'-GATCGCCGACTACAACTACAA-3'	nt 404 – 537 (134 bp)
RBD-P	5'-/56-FAM/AACAGCAAC/ZEN/AACCTGGACAGCAAG/3IBkFQ/-3'	
RBD-R	5'-TCAGGTTGCTCTTCCTGAAC-3'	

Table S4. Sequences of primers and probes designed specific to respective codon-modified vaccine mRNA sequence for duplex ddPCR to quantify the integrity of Moderna mRNA-1273 (Spikevax) vaccine mRNA in plasma (data shown in Figure 4A)

Primer or probe	Sequence	Target region	mRNA integrity region
mRNA1273-1-F	5'-GACCTTCCTGCTGAAGTACAA-3'	nt 876 – 1010	nt 876 – 2474 (1599 bp)
mRNA1273-1-P	5'-/56-FAM/ACCAAGTGC/ZEN/ACCCTGAAGAGCTTC/3IABkFQ/-3'		
mRNA1273-1-R	5'-AAGTTGCTGGTCTGGTAGATG-3'		
mRNA1273-2-F	5'-GTGGAGCAGGACAAGAACA-3'	nt 2370 – 2474	
mRNA1273-2-P	5'-/56-HEX/CGCCCAGGT/ZEN/GAAGCAGATCTACAA/3IBkFQ/-3'		
mRNA1273-2-R	5'-AGGATCTGGCTGAAGTTGAAG-3'		

Table S5. Sequences of primers and probes designed specific to respective codon-modified vaccine mRNA sequence for duplex ddPCR to quantify the integrity of Pfizer/BioNTech BNT162b2 (Comirnaty) vaccine mRNA in plasma (data shown in Figure 4B)

Primer or probe	Sequence	Target Region	mRNA integrity region
BNT-1-F	5'-CAGCTACCAGACACAGACAAA-3'	nt 2070 – 2192	nt 2070 – 3473 (1404 bp)
BNT-1-P	5'-/56-FAM/AGCCAGAGC/ZEN/ATCATTGCCTACACA/3IBkFQ/-3'		
BNT-1-R	5'-GCGATAGAGTTGTTGGAGTAGG-3'		
BNT-2-F	5'-GTGACACAGCGGAACTTCTA-3'	nt 3364 – 3473	
BNT-2-P	5'-/56-HEX/TCATCACCA/ZEN/CCGACAACACCTTCG/3IBkFQ/-3'		
BNT-2-R	5'-GGGTCGTACACGGTATTGTT-3'		

Table S6. Ten two-primer fragments used in duplex PCR assay to detect the degradation patterns of Moderna mRNA-1273 (Spikevax) vaccine mRNA in plasma (data shown in Figure 7A,B)

Fragment	Primer or probe	Sequence	Target region	mRNA integrity region
1	Deg-1-F	5'-CATCATCAGAGGCTGGATCTTC-3'	nt 296 – 405	nt 296 – 1010 (715 bp)
	Deg-1-P	5'-/56-FAM/CACACTGGA/ZEN/CAGCAAGACCCAGAG/3IABkFQ/-3'		
	Deg-1-R	5'-GAACTGGAACTCGCACACTT-3'		
	mRNA1273-1-F	5'-GACCTTCCTGCTGAAGTACAA-3'	nt 876 – 1010	
	mRNA1273-1-P	5'-/56-HEX/ACCAAGTGC/ZEN/ACCCTGAAGAGCTTC/3IABkFQ/-3'		
	mRNA1273-1-R	5'-AAGTTGCTGGTCTGGTAGATG-3'		
2	Deg-1-F	5'-CATCATCAGAGGCTGGATCTTC-3'	nt 296 – 405	nt 296 – 1731 (1436 bp)
	Deg-1-P	5'-/56-FAM/CACACTGGA/ZEN/CAGCAAGACCCAGAG/3IABkFQ/-3'		
	Deg-1-R	5'-GAACTGGAACTCGCACACTT-3'		
	Deg-2-F	5'-CACCAACCTGGTGAAGAACA-3'	nt 1647 – 1731	
	Deg-2-P	5'-/56-HEX/CGTGCTGAC/ZEN/CGAGAGCAACAAGAA/3IABkFQ/-3'		
	Deg-2-R	5'-CTGCTGAAAGGGCAGGAAT-3'		
3	Deg-1-F	5'-CATCATCAGAGGCTGGATCTTC-3'	nt 296 – 405	nt 296 – 2474 (2179 bp)
	Deg-1-P	5'-/56-FAM/CACACTGGA/ZEN/CAGCAAGACCCAGAG/3IABkFQ/-3'		

	Deg-1-R	5'-GAACTGGAACTCGCACACTT-3'		
	mRNA1273-2-F	5'-GTGGAGCAGGACAAGAACA-3'	nt 2370 – 2474	
	mRNA1273-2-P	5'-/56-HEX/CGCCCAGGT/ZEN/GAAGCAGATCTACAA/3IBkFQ/-3'		
	mRNA1273-2-R	5'-AGGATCTGGCTGAAGTTGAAG-3'		
4	mRNA1273-1-F	5'-GACCTTCCTGCTGAAGTACAA-3'	nt 876 – 1010	nt 876 – 1731 (856 bp)
	mRNA1273-1-P	5'-/56-HEX/ACCAAGTGC/ZEN/ACCCTGAAGAGCTTC/3IABkFQ/-3'		
	mRNA1273-1-R	5'-AAGTTGCTGGTCTGGTAGATG-3'		
	Deg-2-F	5'-CACCAACCTGGTGAAGAACA-3'	nt 1647 – 1731	
	Deg-2-P	5'-/56-FAM/CGTGCTGAC/ZEN/CGAGAGCAACAAGAA/3IABkFQ/-3'		
	Deg-2-R	5'-CTGCTGAAAGGGCAGGAAT-3'		
5	Deg-2-F	5'-CACCAACCTGGTGAAGAACA-3'	nt 1647 – 1731	nt 1647 – 2474 (828 bp)
	Deg-2-P	5'-/56-FAM/CGTGCTGAC/ZEN/CGAGAGCAACAAGAA/3IABkFQ/-3'		
	Deg-2-R	5'-CTGCTGAAAGGGCAGGAAT-3'		
	mRNA1273-2-F	5'-GTGGAGCAGGACAAGAACA-3'	nt 2370 – 2474	
	mRNA1273-2-P	5'-/56-HEX/CGCCCAGGT/ZEN/GAAGCAGATCTACAA/3IBkFQ/-3'		
	mRNA1273-2-R	5'-AGGATCTGGCTGAAGTTGAAG-3'		

6	mRNA1273-1-F	5'-GACCTTCCTGCTGAAGTACAA-3'	nt 876 – 1010	nt 876 – 2474 (1599 bp)
	mRNA1273-1-P	5'-/56-FAM/ACCAAGTGC/ZEN/ACCCTGAAGAGCTTC/3IABkFQ/-3'		
	mRNA1273-1-R	5'-AAGTTGCTGGTCTGGTAGATG-3'		
	mRNA1273-2-F	5'-GTGGAGCAGGACAAGAACA-3'	nt 2370 – 2474	
	mRNA1273-2-P	5'-/56-HEX/CGCCCAGGT/ZEN/GAAGCAGATCTACAA/3IBkFQ/-3'		
	mRNA1273-2-R	5'-AGGATCTGGCTGAAGTTGAAG-3'		
7	mRNA1273-1-F	5'-GACCTTCCTGCTGAAGTACAA-3'	nt 876 – 1010	nt 876 – 3477 (2602 bp)
	mRNA1273-1-P	5'-/56-FAM/ACCAAGTGC/ZEN/ACCCTGAAGAGCTTC/3IABkFQ/-3'		
	mRNA1273-1-R	5'-AAGTTGCTGGTCTGGTAGATG-3'		
	Deg-3-F	5'-GTGACACAGCGGAACTTCTA-3'	nt 3367 – 3477	
	Deg-3-P	5'-/56-HEX/TCATCACCA/ZEN/CCGACAACACCTTCG/3IABkFQ/-3'		
	Deg-3-R	5'-GGGTCGTACACGGTATTGTT-3'		
8	Deg-2-F	5'-CACCAACCTGGTGAAGAACA-3'	nt 1647 – 1731	nt 1647 – 3477 (1831 bp)
	Deg-2-P	5'-/56-FAM/CGTGCTGAC/ZEN/CGAGAGCAACAAGAA/3IABkFQ/-3'		
	Deg-2-R	5'-CTGCTGAAAGGGCAGGAAT-3'		
	Deg-3-F	5'-GTGACACAGCGGAACTTCTA-3'	nt 3367 – 3477	

	Deg-3-P	5'-/56-HEX/TCATCACCA/ZEN/CCGACAACACCTTCG/3IABkFQ/-3'		
	Deg-3-R	5'-GGGTCGTACACGGTATTGTT-3'		
9	mRNA1273-2-F	5'-GTGGAGCAGGACAAGAACA-3'	nt 2370 – 2474	nt 2370 – 3477 (1108 bp)
	mRNA1273-2-P	5'-/56-FAM/CGCCCAGGT/ZEN/GAAGCAGATCTACAA/3IBkFQ/-3'		
	mRNA1273-2-R	5'-AGGATCTGGCTGAAGTTGAAG-3'		
	Deg-3-F	5'-GTGACACAGCGGAACTTCTA-3'	nt 3367 – 3477	
	Deg-3-P	5'-/56-HEX/TCATCACCA/ZEN/CCGACAACACCTTCG/3IABkFQ/-3'		
	Deg-3-R	5'-GGGTCGTACACGGTATTGTT-3'		
10	Deg-1-F	5'-CATCATCAGAGGCTGGATCTTC-3'	nt 296 – 405	nt 296 – 3477 (3182 bp)
	Deg-1-P	5'-/56-FAM/CACACTGGA/ZEN/CAGCAAGACCCAGAG/3IABkFQ/-3'		
	Deg-1-R	5'-GAACTGGAACTCGCACACTT-3'		
	Deg-3-F	5'-GTGACACAGCGGAACTTCTA-3'	nt 3367 – 3477	
	Deg-3-P	5'-/56-HEX/TCATCACCA/ZEN/CCGACAACACCTTCG/3IABkFQ/-3'		
	Deg-3-R	5'-GGGTCGTACACGGTATTGTT-3'		

Table S7. Optimized MRM parameters for SM-102, ALC-0315, and DLin-MC3-DMA detection in plasma by mass spectrometry

Ionizable Lipid	Precursor m/z	Product m/z	Q1 pre-bias	Collision energy	Q3 pre-bias	Dwell time (ms)
SM-102	710.6	472.4	-40	-38	-23	50
	710.6	454.4	-40	-45	-24	50
	710.6	318.2	-28	-50	-12	50
	710.6	300.2	-40	-48	-20	50
ALC-0315	767.0	694.6	-20	-42	-36	100
	767.0	510.4	-20	-47	-26	100
	767.0	483.4	-50	-41	-32	100
DLin-MC3-DMA	643.0	132.0	-40	-29	-29	100
	643.0	114.0	-34	-37	-26	100
	643.0	87.0	-22	-55	-16	100

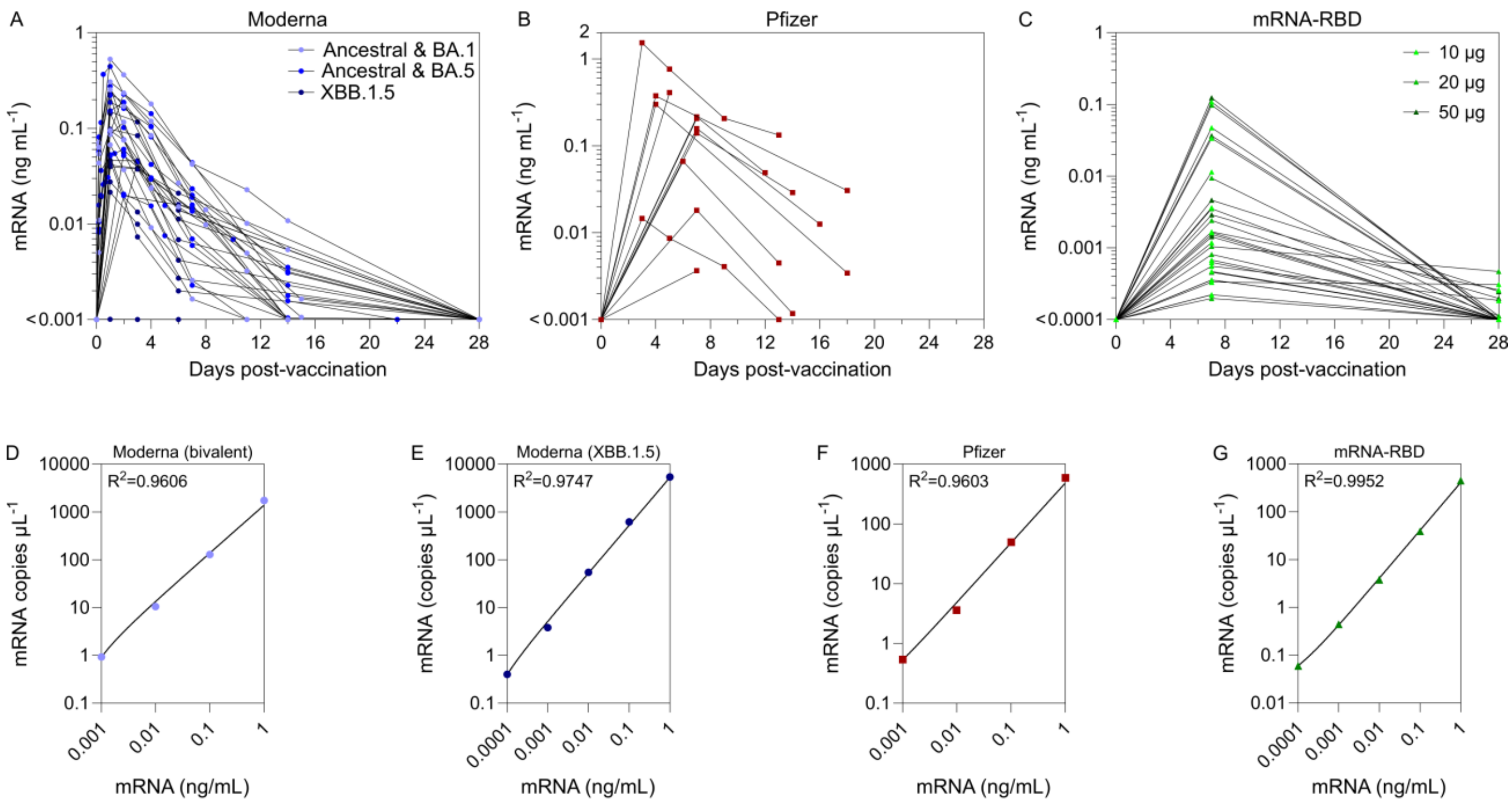

Figure S1. Comparison of *in vivo* vaccine mRNA kinetics in human blood following three types of SARS-CoV-2 mRNA vaccination: Moderna, Pfizer, and mRNA-RBD vaccines. (A–C) Longitudinal vaccine mRNA concentrations (ng mL^{-1}) in human blood from seven cohorts who received either (A) Moderna bivalent ancestral + BA.1, bivalent ancestral + BA.5, or monovalent XBB.1.5; (B) Pfizer; or (C) mRNA-RBD vaccination at 10, 20, or 50 µg doses. (D–G) Linear PCR standard curves for (D) Moderna bivalent, (E) Moderna XBB.1.5, (F) Pfizer, and (G) mRNA-RBD vaccine mRNA in human plasma, which were used to determine the lower limit of quantification (LLOQ) shown in Fig. 2A–C and to convert mRNA concentration from copies μL^{-1} to ng mL^{-1}. Samples for the linear standard curves were prepared by spiking Moderna bivalent, Moderna XBB.1.5, Pfizer, or mRNA-RBD vaccines (with known mRNA concentrations) into human plasma (collected from one donor before vaccination), followed by RNA extraction and reverse transcription. The resulting cDNA was serially diluted 10-fold in nuclease-free water and analyzed by ddPCR.

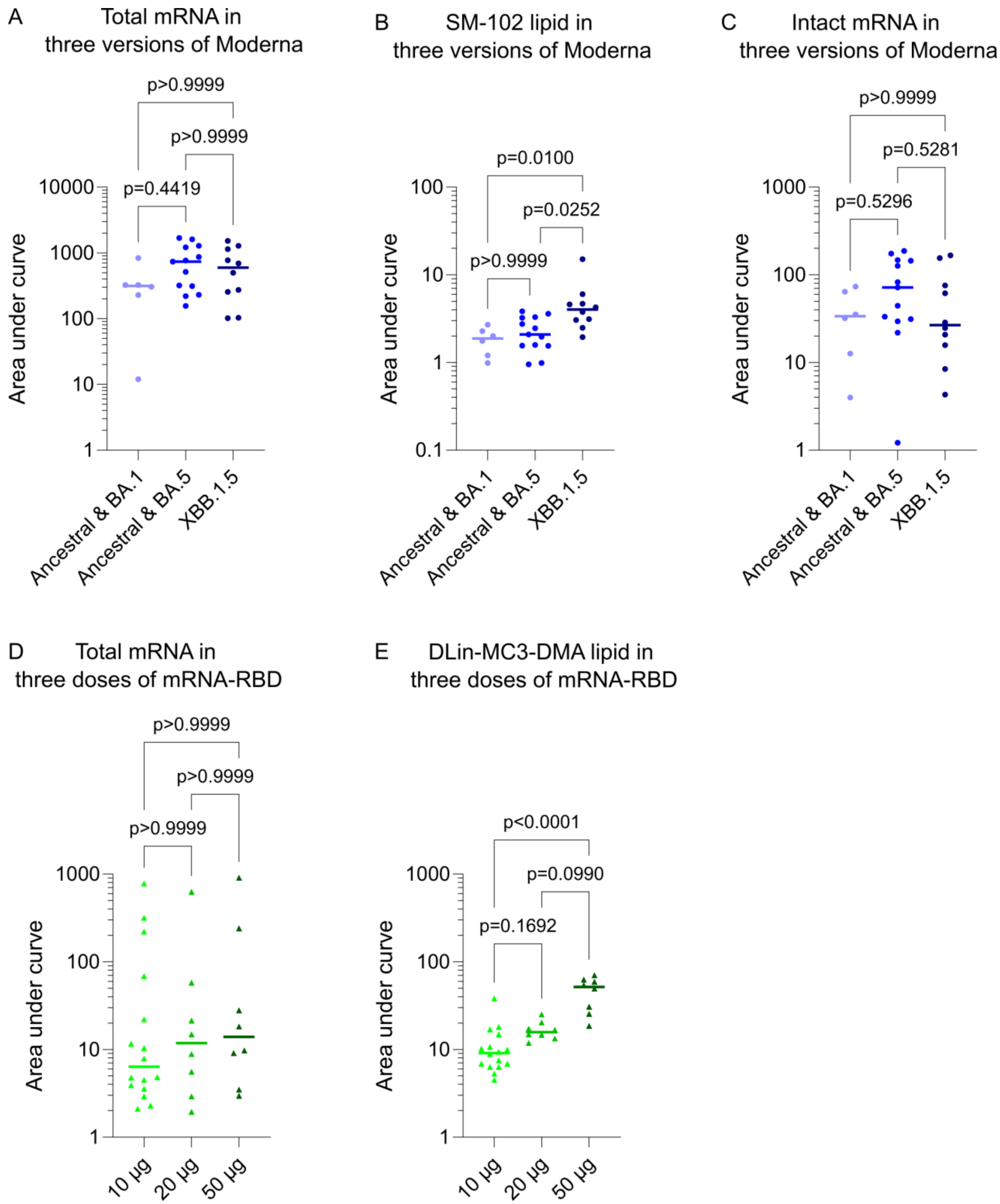

Figure S2. Blood distribution of three versions of the Moderna vaccine (bivalent ancestral + BA.1, bivalent ancestral + BA.5, or monovalent XBB.1.5) and three doses of the mRNA-RBD vaccine (10, 20, 50 µg). (A,D) Total mRNA, (B,E) ionizable lipid, and (C) intact mRNA exposure in blood,

expressed as model-derived post-peak AUCs corresponding to the analyses in Figures 2F, 3F, and 4F, respectively. In (A–E), each dot represents one participant, and the horizontal line indicates the median. Statistical analysis was performed using the nonparametric Kruskal–Wallis test with Dunn's multiple comparisons in (A–E).

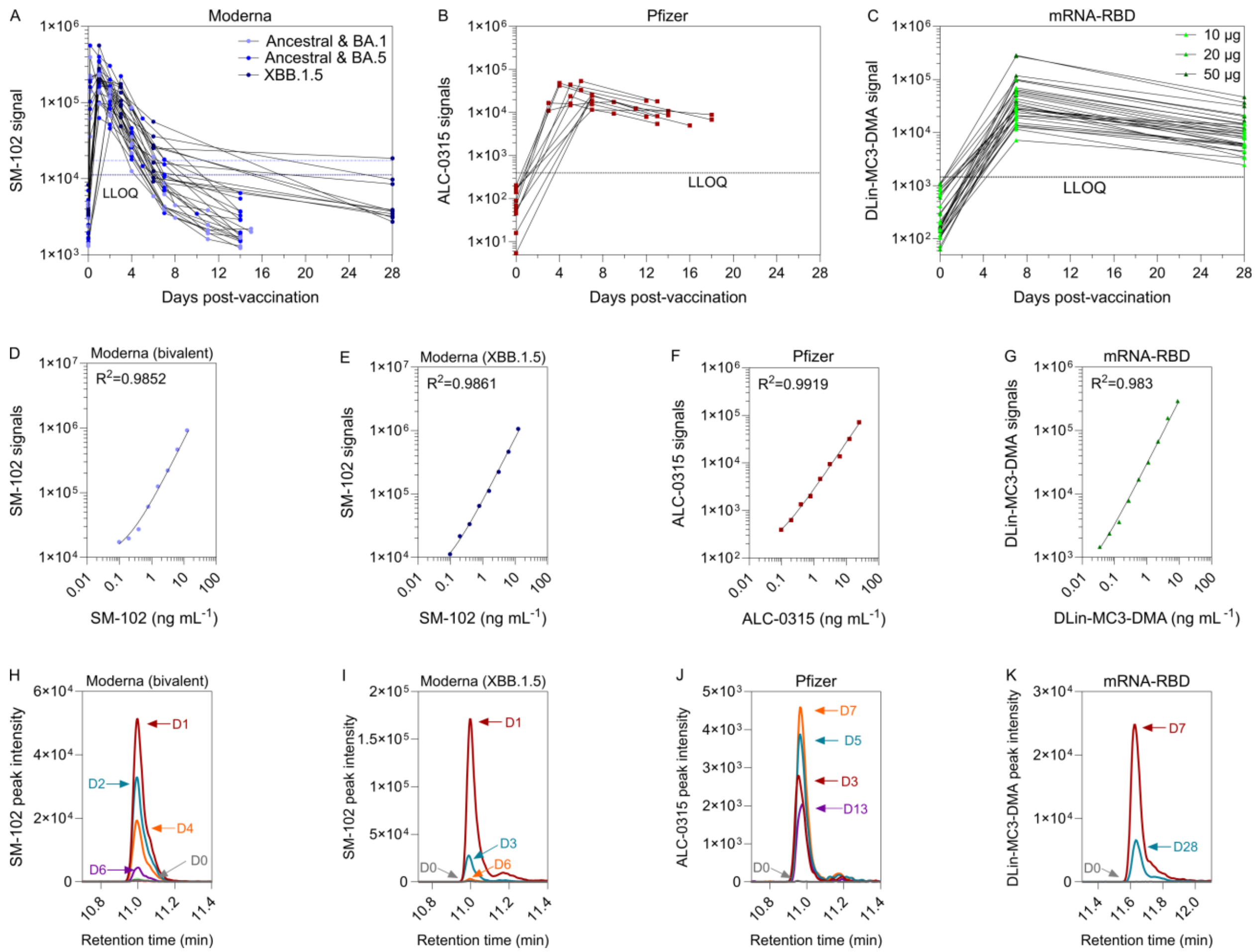

Figure S3. Raw mass spectrometry data showing *in vivo* ionizable lipid kinetics in human blood following Moderna, Pfizer, or mRNA-RBD vaccination. (A–C) Longitudinal raw ionizable lipid signals in human blood from seven cohorts who received either (A) Moderna bivalent ancestral + BA.1, bivalent ancestral + BA.5, or monovalent XBB.1.5 (formulated with SM-102); (B) Pfizer (formulated with ALC-0315); or (C) mRNA-RBD (formulated with DLin-MC3-DMA) vaccination at 10, 20, or 50 µg doses. In panel A, two LLOQs are shown: 11207 for Moderna XBB.1.5 (dark blue dashed line) and 17257 for Moderna bivalent vaccines (light blue dashed line). (D–G) Linear standard curves for (D) SM-102 from Moderna bivalent vaccine, (E) SM-102 from Moderna XBB.1.5 vaccine, (F) ALC-0315 from Pfizer vaccine, and (G) DLin-MC3-DMA from mRNA-RBD vaccine in human plasma, which were used to determine the LLOQ and to convert

raw lipid signals to concentrations (ng mL^{-1}) shown in Figure 3A–C. Samples for the linear standard curves were prepared by titrating Moderna bivalent, Moderna XBB.1.5, Pfizer, or mRNA-RBD vaccines (with known ionizable lipid concentration) into human plasma (from one donor collected pre-vaccination) at varying ionizable lipid concentrations. (H–K) Representative chromatograms showing the peak intensity of (H,I) SM-102, (J) ALC-0315, and (K) DLin-MC3-DMA lipid signals in longitudinal plasma samples from individuals who received (H) Moderna bivalent, (I) Moderna XBB.1.5, (J) Pfizer, or (K) mRNA-RBD vaccination, as determined by liquid chromatography–mass spectrometry (LC–MS).

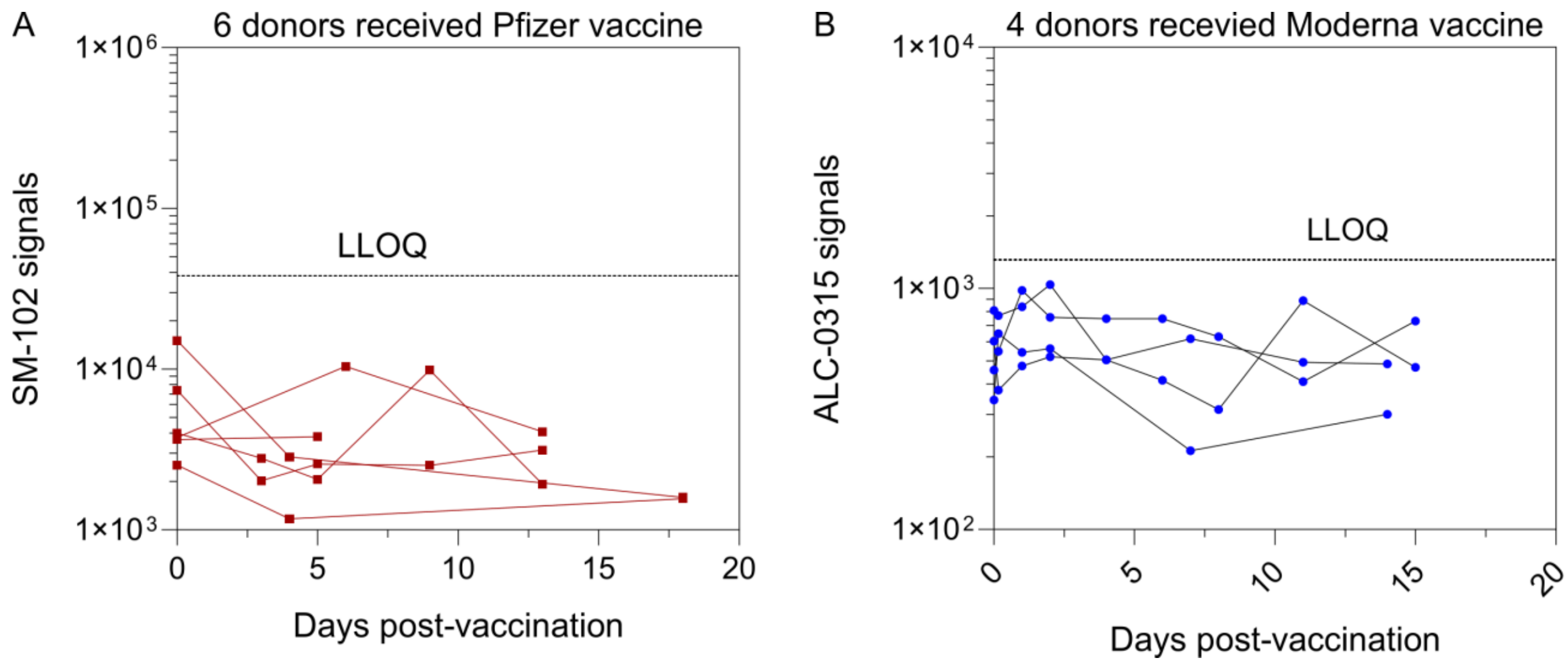

Figure S4. Control experiment demonstrating the high specificity of the lipidomics analysis. (A) No SM-102 lipid signals were detected above the LLOQ in longitudinal plasma samples from six donors who received Pfizer vaccination. (B) No ALC-0315 lipid signals were detected above LLOQ in longitudinal plasma samples from four donors who received Moderna vaccination. The LLOQ was 0.1 ng mL^{-1} for both SM-102 and ALC-0315, as determined from their respective linear standard curves.

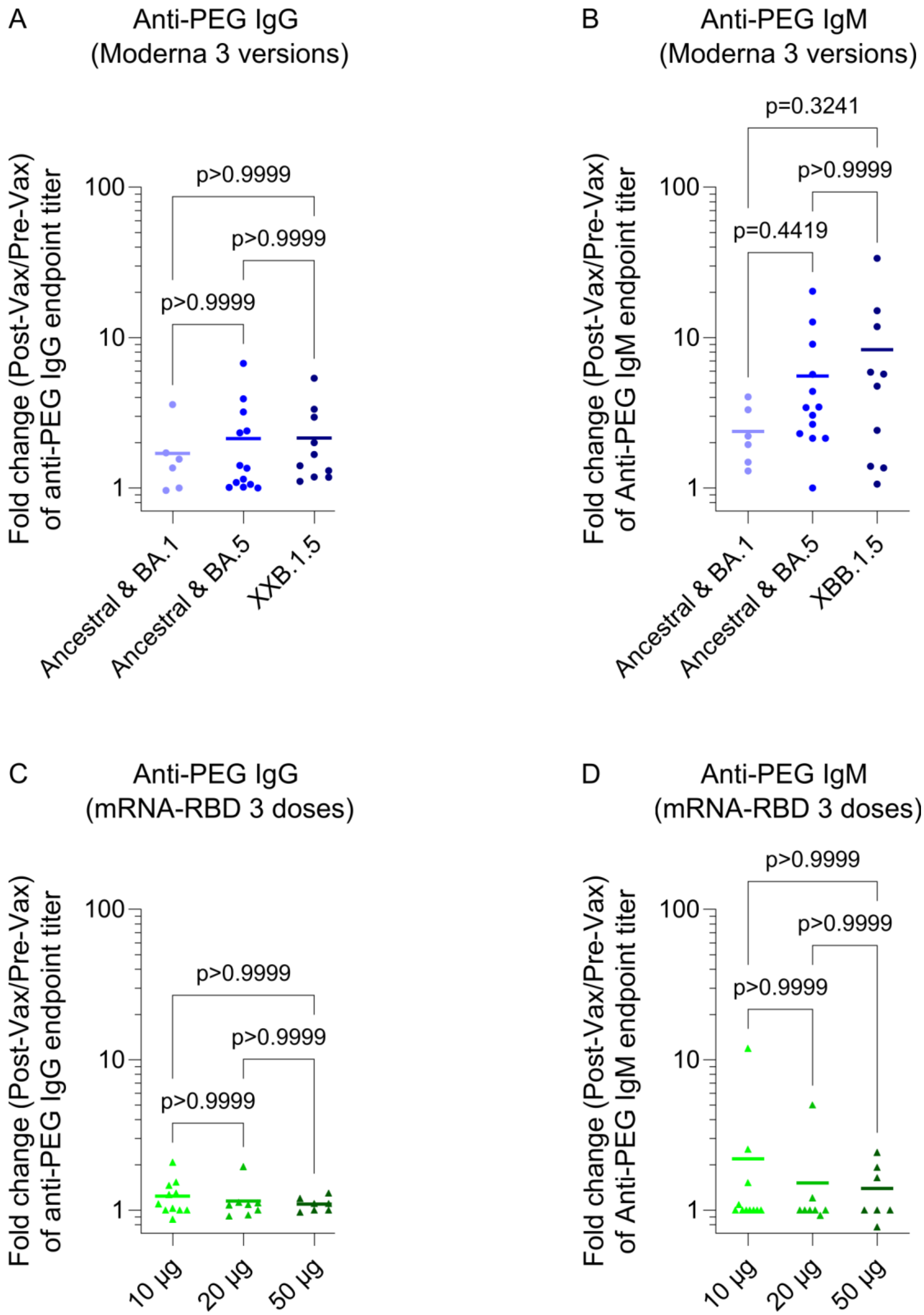

Figure S5. Comparison of anti-PEG antibody boosting across three versions of the Moderna vaccine (bivalent ancestral + BA.1, bivalent ancestral + BA.5, and monovalent XBB.1.5) and three

doses of the mRNA-RBD vaccine (10, 20, or 50 μg). (A,B) Comparison of fold changes in anti-PEG IgG and IgM titers among the three Moderna vaccine versions. (C,D) Comparison of fold changes in anti-PEG IgG and IgM titers among the three mRNA-RBD vaccine dose groups. In (A–D), each dot represents one participant, and the horizontal line indicates the mean. Statistical analysis was performed using the nonparametric Kruskal–Wallis test with Dunn's multiple comparisons in (A–D).

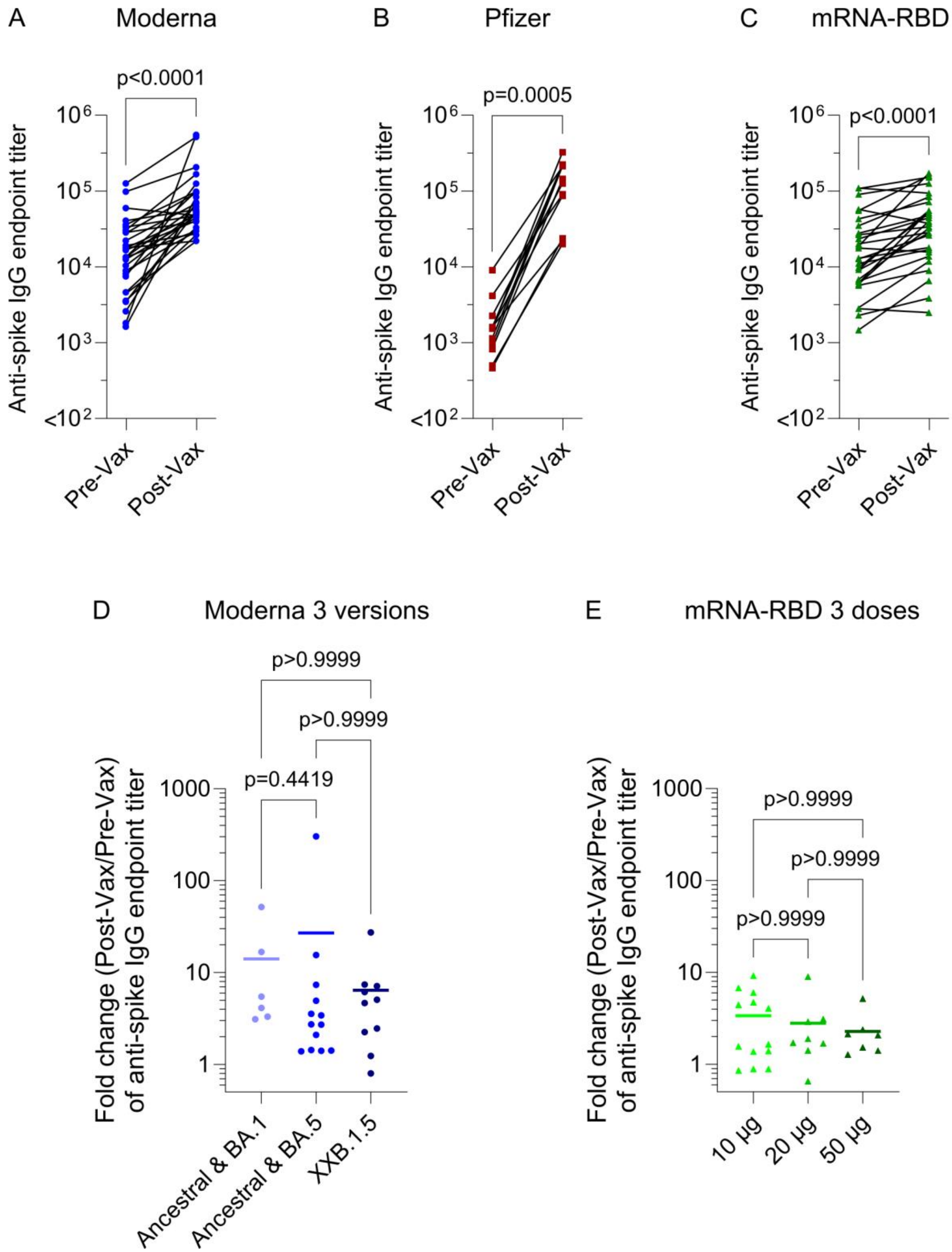

Figure S6. Comparison of anti-spike antibody levels in human blood before and after Moderna, Pfizer, or mRNA-RBD vaccination. (A–C) Comparison of plasma anti-spike IgG titers before vaccination (Pre-Vax) and after vaccination (Post-Vax) for the three vaccine platforms. (D)

Comparison of fold changes in anti-spike IgG titers among the Moderna vaccine versions. (E) Comparison of fold changes in anti-spike IgG titers among the three mRNA-RBD vaccine dose groups. In (D,E), each dot represents one participant, and the horizontal line indicates the mean. Statistical analysis was performed using the nonparametric Wilcoxon matched-pairs signed rank test in (A–C) and the nonparametric Kruskal–Wallis test with Dunn’s multiple comparisons in (D,E).
